# Targeting ORMDL2 in glioblastoma through integrated landscape of high-throughput sequencing and pharmacogenomic analysis

**DOI:** 10.7150/ijms.116954

**Published:** 2025-09-27

**Authors:** Fitria Sari Wulandari, Chih-Yang Wang, Sachin Kumar, Ngoc Phung Ly, Juan Lorell Ngadio, Dahlak Daniel Solomon, Do Thi Minh Xuan, Gabriela Natasha Sutandi, Hung-Yun Lin, Hui-Ru Lin, Yung-Kuo Lee, Hao-Chien Yang, Ming-Cheng Tsai, Ching-Chung Ko

**Affiliations:** 1 Graduate Institute of Cancer Biology and Drug Discovery, College of Medical Science and Technology, Taipei Medical University, Taipei 11031, Taiwan.; 2 Department of Surgery, Division of Neurosurgery, Shuang Ho Hospital, Taipei Medical University, New Taipei City, Taiwan.; 3 Ph.D. Program for Cancer Molecular Biology and Drug Discovery, College of Medical Science and Technology, Taipei Medical University, Taipei 11031, Taiwan.; 4 TMU Research Center of Cancer Translational Medicine, Taipei Medical University, Taipei 11031, Taiwan.; 5 Faculty of Applied Sciences and Biotechnology, Shoolini University of Biotechnology and Management Sciences, Himachal Pradesh, 173229, India.; 6 Department of Bioinformatics, School of Life Sciences, Indonesia International Institute for Life Sciences, Jl. Pulomas Barat Kav 88, Jakarta Timur, 13210, Indonesia.; 7 Yogananda School of AI Computers and Data Sciences, Shoolini University, Solan 173229, India.; 8 Faculty of Pharmacy, Van Lang University, 69/68 Dang Thuy Tram Street, Binh Loi Trung Ward, Ho Chi Minh City 70000, Vietnam.; 9 Division of Biology and Bioinformatics, School of Bioscience, University of Skövde, Högskolevägen 1, PO Box 408, 541 28 Skövde, Sweden.; 10 Cancer Center, Wan Fang Hospital, Taipei Medical University, Taipei 11031, Taiwan.; 11 Traditional Herbal Medicine Research Center of Taipei Medical University Hospital, Taipei Medical University, Taipei 11031, Taiwan.; 12 Pharmaceutical Research Institute, Albany College of Pharmacy and Health Sciences, Rensselaer, NY 12144, USA.; 13 Institute of Medical Science and Technology, National Sun Yat-Sen University, Kaohsiung 80424, Taiwan.; 14 Nursing Department, Kaohsiung Armed Forces General Hospital, Kaohsiung 80284, Taiwan.; 15 Division of Experimental Surgery Center, Department of Surgery, Tri-Service General Hospital, National Defense Medical University, Taipei 11490, Taiwan.; 16 Medical laboratory, Medical Education and Research Center, Kaohsiung 80284, Taiwan.; 17 School of Medicine, Fu Jen Catholic University, New Taipei City 242, Taiwan.; 18 Department of Neurosurgery, Shin-Kong Wu Ho-Su Memorial Hospital, 95 Wen-Chang Road, Shih-Lin District, Taipei 111045, Taiwan.; 19 Department of Medical Imaging, Chi Mei Medical Center, Tainan, 71004, Taiwan.; 20 Department of Health and Nutrition, Chia Nan University of Pharmacy and Science, Tainan, 71710, Taiwan.; 21 School of Medicine, College of Medicine, National Sun Yat-Sen University, Kaohsiung, 80424, Taiwan.

**Keywords:** Multi-omics Analysis, Tumor Microenvironment (TME), Glioblastoma, ORMDL2, Drug Discovery

## Abstract

Glioblastoma multiforme (GBM) is characterized by rapid progression, therapeutic resistance, and a profoundly immunosuppressive tumor microenvironment. Emerging evidence suggests that endoplasmic reticulum (ER)-associated macromolecules play critical roles in tumor adaptation. In this study, we performed a multi-omics investigation of orosomucoid-like protein 2 (ORMDL2), a conserved ER membrane protein involved in sphingolipid biosynthesis and ER stress regulation, and uncovered its regulatory functions in GBM progression. Transcriptomic analyses across The Cancer Genome Atlas (TCGA), and Chinese Glioma Genome Atlas (CGGA) revealed elevated ORMDL2 expression in GBM tissues which causes poor prognosis. The MetaCore pathway and Gene Set Enrichment Analysis (GSEA) identified ORMDL2's involvement in antigen presentation via a major histocompatibility complex I (MHC class I), unfolded protein response (UPR), and mitochondrial apoptotic signaling. Single-cell RNA-sequencing data and the Human Protein Atlas showed ORMDL2 enrichment in tumor stromal cells. Pharmacogenomic correlation via the Genomics in Drug Sensitivity in Cancer (GDSC) and Cancer Therapeutics Response Portal (CTRP) database suggested that ORMDL2 expression was associated with resistance to DNA damage response inhibitors such as etoposide, doxorubicin, talazoparib, and might interact with sphingolipid-targeting compounds. Collectively, our findings establish ORMDL2 as a multi-functional macromolecular regulator of immune suppression and therapeutic resistance in GBM, providing new mechanistic insights and potential targets for translational medicines.

## Introduction

Glioblastoma multiforme (GBM) is the most aggressive and lethal form of primary malignant brain tumors in adults, accounting for approximately 15% of all intracranial neoplasms and over 45% of malignant gliomas 1, 2. Despite aggressive treatment strategies that combine surgical resection, radiotherapy, and chemotherapy with temozolomide (TMZ), GBM exhibits a high recurrence rate, therapy resistance, and a poor prognosis, with a median survival of only 12-15 months. Hallmarks of GBM pathogenesis include pervasive genomic instability, cellular plasticity, an aberrant DNA damage response (DDR), and a profoundly immunosuppressive tumor microenvironment (TME) 3. These features collectively contribute to the failure of current treatment regimens, and highlight the need to identifying novel molecular targets capable of simultaneously addressing multiple therapeutic vulnerabilities simultaneously 4-6. Among the key regulators of tumor behavior in GBM are biological macromolecules, such as RNA-binding proteins, metabolic enzymes, and membrane-bound signaling scaffolds, that coordinate fundamental processes including stress adaptation, lipid metabolism, immune evasion, and DNA repair. Identifying such macromolecular drivers is crucial for understanding GBM progression at the systems level 7-9.

In this context, the orosomucoid-like (ORMDL) family of transmembrane proteins has garnered attention due to its regulatory roles in lipid metabolism and endoplasmic reticular (ER) stress responses 10. ORMDL protein 2 (ORMDL2) also named ORMDL sphingolipid biosynthesis regulator 2 (ORMDL2), is one of the three mammalian paralogs in this family of highly conserved ER-localized macromolecules that controls sphingolipid biosynthesis through feedback inhibition of serine palmitoyl transferase (SPT), the rate-limiting enzyme in ceramide production 11-13. Sphingolipids are structurally complex biological macromolecules that serve as essential regulators of membrane architecture, signal transduction, and cellular fate. Ceramides and their derivatives, including sphingosine-1-phosphate (S1P), orchestrate a range of oncogenic processes such as proliferation, apoptosis, immune suppression, and treatment resistance. Dysregulation of sphingolipid metabolism has been implicated in various cancers, yet the specific function of ORMDL2 in glioblastoma remains poorly defined. Preliminary studies in other systems suggested that ORMDL2 can modulate inflammation, oxidative stress, and unfolded protein responses (UPRs), processes that are critically involved in GBM's pathobiology. However, no comprehensive multi-omics analysis has been conducted to evaluate ORMDL2's expression, regulation, or downstream impact in GBM.

**Figure 1:**
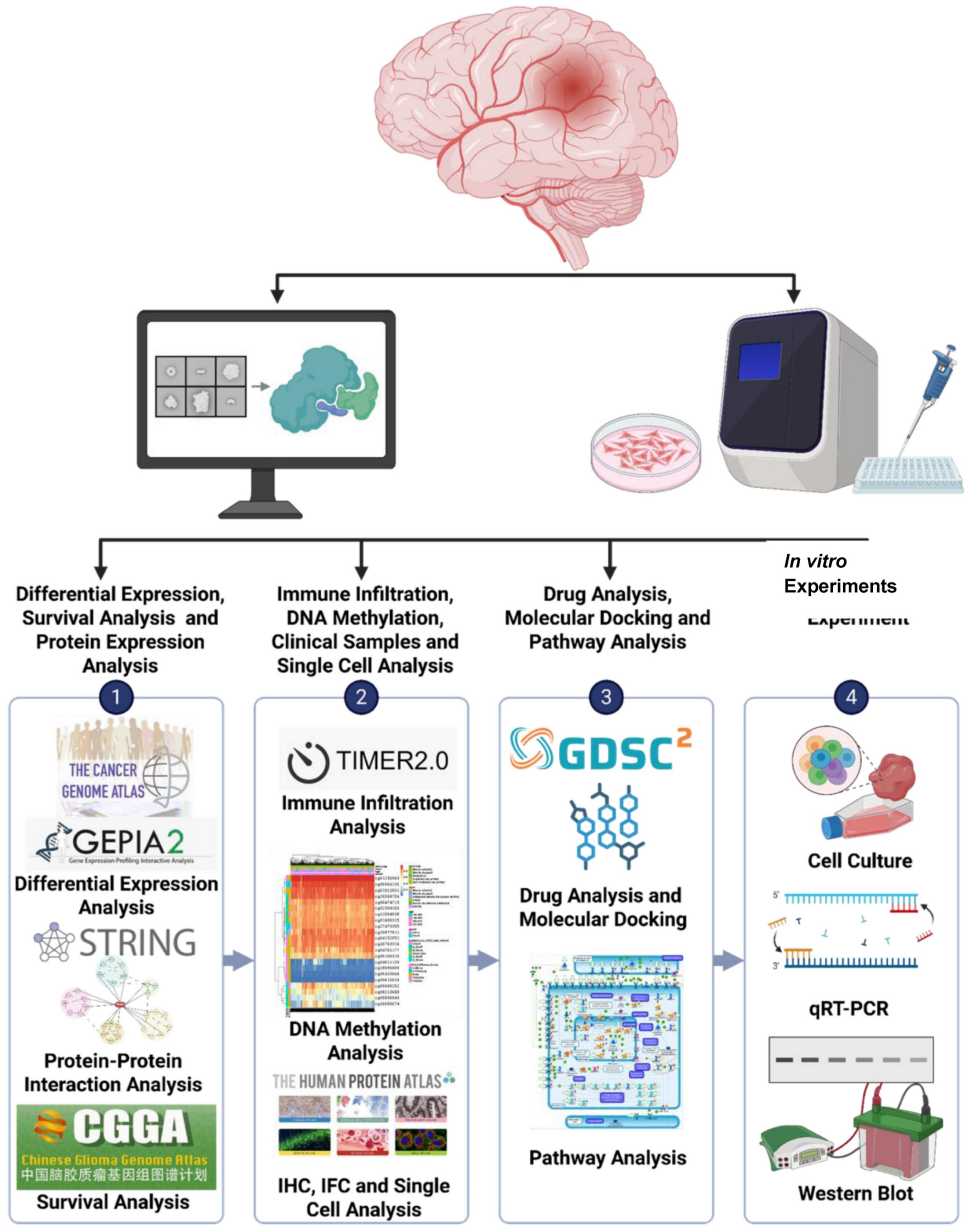
** Flowchart of the study design and analytical process for GBM**: Gene expression data were obtained from publicly accessible databases, such as TCGA, CGGA, GTEx, etc. to analyze the expression levels of genes in GBM tissues compared to normal brain tissues and explore their potential association with patient prognoses.

In this study, we identified the ORMDL2-encoded protein, ORM1-like protein 2, as a novel ER-localized macromolecule involved in GBM progression, integrating metabolic reprogramming, epigenetic activation, and immune remodeling. Transcriptomic analyses from The Cancer Genome Atlas (TCGA) and Chinese Glioma Genome Atlas (CGGA) confirmed its overexpression in GBM and association with poor survival. DNA hypomethylation at its promoter region suggested epigenetic derepression. Co-expression and pathway enrichment analyses revealed ORMDL2's link to oxidative phosphorylation, ER stress, and ceramide biosynthesis. Single-cell RNA-sequencing (scRNA-Seq) data further demonstrated its enrichment in different cells, implying a role in immune evasion and therapy resistance. A pharmacogenomic analysis showed that ORMDL2 was correlated with resistance to DNA-damaging agents and poly(ADP ribose) polymerase (PARP) inhibitors 14, 15, while molecular docking suggested druggable interaction sites at the ORMDL2-SPT axis. Functional knockdown in A172 cells supported its role in proliferation, migration, and TMZ sensitivity 15-17. Collectively, our integrative approach (Fig. 1) revealed ORMDL2 as a key immune and metabolic modulator and promising therapeutic target in GBM.

## Materials and Methods

### Pan-cancer transcriptomic profiling and glioma subtype analysis

To determine ORMDL2's expression across various malignancies, we accessed normalized RNA-Seq data from TCGA and Genetype-Tissue Expression (GTEx) via UCSC Xena. Comparative analysis across 33 cancer types showed GBM to be one of the highest ORMDL2-expressing tumors. Expression differences between GBM and normal brain tissues were evaluated via Gene Expression Profiling Interactive Analysis 2 (GEPIA2), while UALCAN provided a subgroup analysis by histological grade 18-20. We employed the clusterProfiler R package 21, and SRplot platform 22 to conducting functional enrichment analyses 23-25. An International Prognostic Score (IPS) analysis (https://tcia.at/home) was used to explore the gene expressions of specific immune-related gene sets, cellular compositions of immune infiltrates characterized using gene set enrichment analyses (GSEAs) and deconvolution, neoantigens and cancer-germline antigens 26, Human Leukocyte Antigen (HLA) types, and tumor heterogeneity estimated from cancer cell fractions 27-31.

### Survival and DNA methylation analysis

CGGA and TCGA-GBM clinical cohorts were used to investigate the prognostic impact of ORMDL2. A Kaplan-Meier analysis stratified patients by median expression levels, and a Cox proportional hazards regression was applied for both univariate and multivariate models which were adjusted for age, isocitrate dehydrogenase (IDH) status, and therapy. Prognostic nomograms were constructed using the rms package, and time-dependent receiver operating characteristic (ROC) curves were plotted to evaluate predictive performance 32-35. Meanwhile, a DNA methylation analysis was conducted to examine variations in methylation patterns of ORMDL2 in GBM patients using data from the TCGA dataset. To achieve this, we utilized several methylation databases by MethSurv 36-40.

### Pathway enrichment, co-expression network, and immune module identification

We used LinkedOmics and Omics Playground v.3.4.1 41 to identify the top 25% of genes co-expressed with ORMDL2 across the TCGA-GBM cohort. These genes were subjected to Gene Ontology (GO) and Kyoto Encyclopedia Genes and Genome (KEGG) pathway enrichment analyses using cluster Profiler. Functional modules related to sphingolipid metabolism, UPRs, and mitochondrial apoptosis were enriched. Furthermore, MetaCore was employed to construct networked pathways derived from an input gene list to explore biological processes as previously described 42.

### Single-cell RNA-Seq (scRNA-Seq) and tumor subpopulation mapping

To further investigate ORMDL2's role in different cell types within the GBM tumor microenvironment, scRNA-Seq data from publicly available GBM datasets were analyzed. Using the Human Protein Atlas (HPA) dataset 43 and CancerSCEM 44, single-cell clusters were identified, and ORMDL2 expression was assessed across various cell types, including glioma stem cells, neurons, astrocytes, microglial cells, and endothelial cells. The t-Distributed stochastic neighbor embedding (t-SNE) and Uniform Manifold Approximation and Projection (UMAP) techniques were applied for dimensionality reduction and visualization of cell clusters based on gene expressions 45-48.

### Drug sensitivity and molecular docking analysis

Drug targets from Gene Set Cancer Analysis (GCSA; Cancer Therapeutic Response Portal (CTRP) and Genomics of Drug Sensitivity in Cancer (GDSC)) 49 alongside as phosphatidylinositol 3-kinase (PI3K) 50 and histone deacetylase (HDAC) inhibitors 51 were tested for their efficacy. Drug targets from the GSCA platform including afanitib and lapatinib with PI3K inhibitor including Copanlisib, Idelalisib, Umbralisib, Duvelisib, and Alpelisib while the tested HDAC inhibitor were vorinostat and phenylbutyrate 52-56. The SDF structure files of these drugs were then retrieved from PubChem (https://pubchem.ncbi.nlm.nih.gov/). Energy minimization of the ligand used Avogadro 1.20 with the MMFF94 force filed and steepest descent algorithm while AutoDockTools was used to convert to pdbqt file. The protein structure of ORMDL2 was taken from the AlphaFold (https://alphafold.ebi.ac.uk) with the structure ID Q53FV1. The predicted structure from AlphaFold was cleaned by removing low-confidence amino acids following PyMol. The addition of hydrogen bonds, and Gasteiger charges and energy minimization were done in USCS Chimera software 1.18. The addition of Gasteiger used AMBER ff14SB while the energy minimization setting was on default. The protein was then converted to pdbqt format in AutoDockTools. CastPFold was utilized to determine potential binding sites before being mapped. Docking was then performed using AutoDock Vina with the energy range set to 4 and exhaustiveness set to 8. Three- (3D) and two-dimensional (2D) visualization of the docked structure were then done using Biovia Discovery Studio 2025 57-60.

### Cell culture

Human U87 and A172 GBM cell lines and the patient-derived Pt#3 lines were used, along with their respective TMZ-resistant counterparts of U87R and A172R (with acquired TMZ resistance) and Pt#3R (intrinsically resistant) 61-65. TMZ-resistant cell lines were maintained in a low dose of TMZ of 50μM (MedChemExpress Cat. No: HY-17364, USA). All cell lines were cultured in Dulbecco's modified eagle medium (DMEM) supplemented with 10% fetal bovine serum (FBS) and 1% penicillin-streptomycin at 37 ^o^C in humidified incubator with 5% CO_2._

### Protein extraction and western blot

Cells were washed twice with ice-cold 1X phosphate-buffered saline (PBS), and then lysed in 50 μL of RIPA buffer supplemented with 1% protease and phosphatase inhibitors. After incubation on ice for 10 min, cells were scraped, and lysates were collected into 1.5-mL tubes. Samples were centrifuged at 13,000 rpm for 30 min at 4 °C, and supernatants were stored at -20°C. Protein concentrations were measured using the bicinchoninic acid (BCA) assay. Proteins (30 μg) were denatured at 95 °C for 10 min, separated by 10% sodium dodecylsulfate polyacrylamide gel electrophoresis (SDS-PAGE), and transferred to 0.45 μm polyvinylidene difluoride (PVDF) membranes (Mercks Millipore, USA) at 350 mA for 2 h. Membranes were blocked with 5% non-fat milk in 0.1% Tris-buffered saline Tween-20 (TBST) for 1 h, then incubated overnight at 4 °C with primary antibodies against ORMDL2 (Aviva System Biology #AAP77319 1:500, USA) and GAPDH (Cat. No. AB2302 1:5000; Sigma-Aldrich, St. Louis, MO, USA). After washing, membranes were incubated with horseradish peroxidase (HRP)-conjugated secondary antibodies (1:5000) for 1 h. Signals were detected using a chemiluminescent substrate (T-Pro) and imaged with the e-BLOT Touch Imager (e-BLOT, Shanghai, China) 66-70.

### RNA extraction and reverse transcriptase polymerase chain reaction

Total RNA was extracted using the Trizol reagent (CyrusBioscience, Taiwan). Cells were scraped and transferred to 1.5-mL microcentrifuge tubes. Ice-cold chloroform (200 μL) was added, and samples were vortexed for 20-30 s, then incubated at room temperature for 10 min. After centrifugation at 13,000 rpm for 10 min at 4°C, the aqueous phase was transferred to new tubes and mixed 1:1 with ice-cold isopropanol. Samples were incubated on ice for 30 min, gently inverted, and centrifuged at 14,000 rpm for 5 min. Pellets were washed twice with 75% ethanol, centrifuged between washes, air-dried for 30 min, and resuspended in 20-30 μL of nuclease-free water. RNA was dissolved at 37°C for 10 min or 65°C for 5 min and stored at -80°C. Reverse transcription was performed using the PrimeScript RT Kit (Takara Bio, USA) according to the manufacturer's instructions 71-73.

### Real-time polymerase chain reaction

For the quantitave (q)PCR, 1 μL of complementary (c)DNA was mixed with 5 μL of TB Green Premix, and a separate primer mix was prepared that contained 0.2 μL of the forward primer (10 μM), 0.2 μL of the reverse primer (10 μM), and 3.6 μL of nuclease-free water was prepared. The primer mix (4 μL) was first added to each well of a 96-well plate, followed by the cDNA/TB Green mixture (6 μL), for a total reaction volume of 10 μL/well. Non-template controls (NTCs) were prepared in the same way without cDNA. Plates were sealed, briefly centrifuged, and run on the LightCycler® 96 Real-Time PCR System (Roche, USA). Relative expression levels were analyzed using the 2^-ΔΔCt^ method. Primer sequences for ORMDL2 and GAPDH (as the housekeeping gene) are listed below. For ORMDL2. F: CAGCATTCCTGTTGTCTGGACC and R: TGTCAGTAGCCGAGCCTTTCCT and for GAPDH, F: GTCTCCTCTGACTTCAACAGCG and R: ACCACCCTGTTGCTGTAGCCAA. Expression levels were normalized and calculated using the 2^-^ΔΔCt^ method.

### Statistical analysis

Data were analyzed by GraphPad Prism Software vers. 10 (GraphPad, La Jolla, CA, USA). Statistical differences were evaluated by using an unpaired Student's t-test with a one-tailed distribution multivariable correlation or two-way analysis of variance (ANOVA). Data are presented as the mean ± standard error of the mean (SEM) values. A *p*-value of < 0.05 was interpreted as statistically significant (* p < 0.05; ** p < 0.01; *** p < 0.001; **** p < 0.0001). Experiments were replicated at least thrice.

## Results

### ORMDL2 is overexpressed in GBM and was correlated with poor clinical features

**Figure 2:**
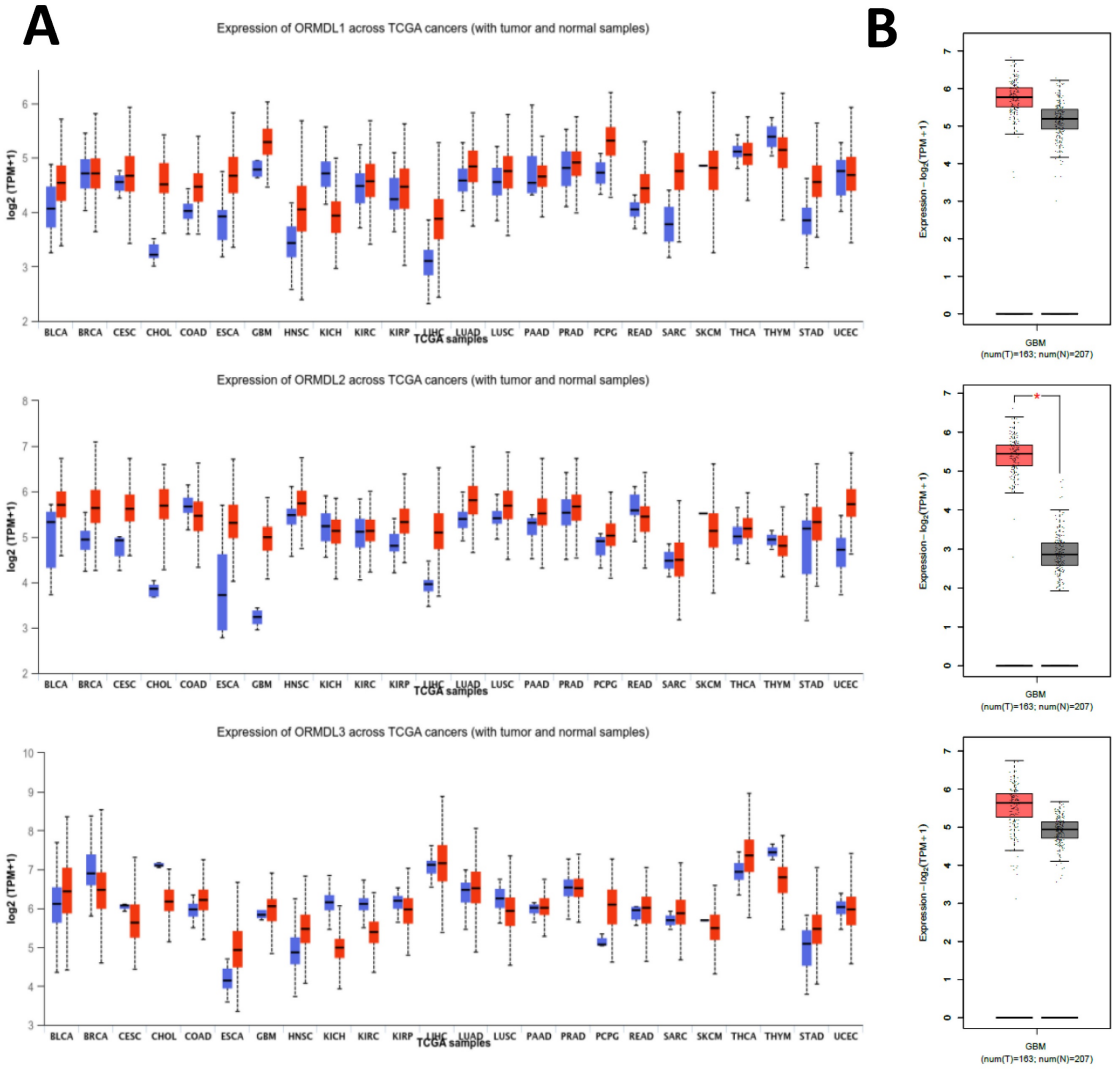
** Expression profiles of ORMDL family genes in GBM and other cancer types.** (A) Box plots showing the expression levels (log₂ TPM+1) of ORMDL1, ORMDL2, and ORMDL3 across various tumor types compared to normal tissues from TCGA datasets. Red boxes represent tumor samples, and blue boxes represent normal tissues. (B) Comparative expressions of ORMDL1, ORMDL2, and ORMDL3 in GBM tissues (n = 163) versus normal brain tissues (n = 207). Among the three, ORMDL2 showed the most significant overexpression in GBM (p < 0.01).

**Figure 3:**
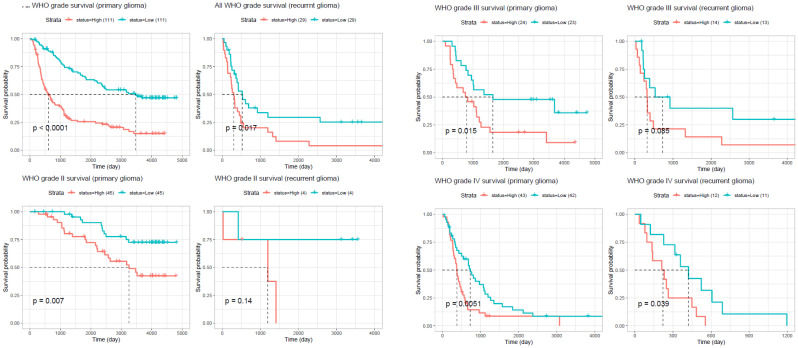
** Kaplan-Meier survival analysis comparing overall survival between glioma patients with high and low ORMDL2 expression across different WHO grades and recurrence status.** In primary gliomas, high ORMDL2 expression was significantly associated with shorter survival (p < 0.0001). Similar trends were observed in recurrent gliomas (p = 0.017), and in specific subgroups including WHO grade III primary (p = 0.015), WHO grade III recurrent (p = 0.0085), and WHO grade IV primary gliomas (p = 0.0051). Although no significant difference was found in WHO grade II recurrent gliomas (p = 0.14), a marginal significance was observed in WHO grade IV recurrent cases (p = 0.039). These findings suggest that elevated ORMDL2 expression may serve as a negative prognostic marker in glioma, particularly in higher-grade and primary tumors.

To investigate the expression profile of the *ORMDL* gene family in cancer, pan-cancer analysis was performed using the GEPIA2 platform across 33 TCGA tumor types and matched normal tissues. Among the three paralogs, *ORMDL2* exhibited the most significant overexpression in GBM, while *ORMDL1* and *ORMDL3* showed less-consistent trends (Fig. 2A). A boxplot analysis further confirmed that *ORMDL2* expression in GBM was significantly higher than that in normal brain tissues (*p* < 0.001, Wilcoxon test) (Fig.2B). In contrast, expressions of *ORMDL1* and *ORMDL3* non-significantly differed between tumor and normal brain tissues. CGGA datasets were also used to support the findings from TCGA datasets (Fig. 3; Supplementary Data S2). Kaplan-Meier survival analyses were stratified by *ORMDL2* expression across multiple subgroups. High *ORMDL2* messenger (m)RNA was associated with poorer overall survival in all primary and recurrent brain tumor patients. CGGA data were also stratified *ORMDL2* mRNA expression by its clinicopathological features (Supplementary Data S1).

### Promoter hypomethylation and chromatin accessibility contribute to ORMDL2 upregulation

**Figure 4:**
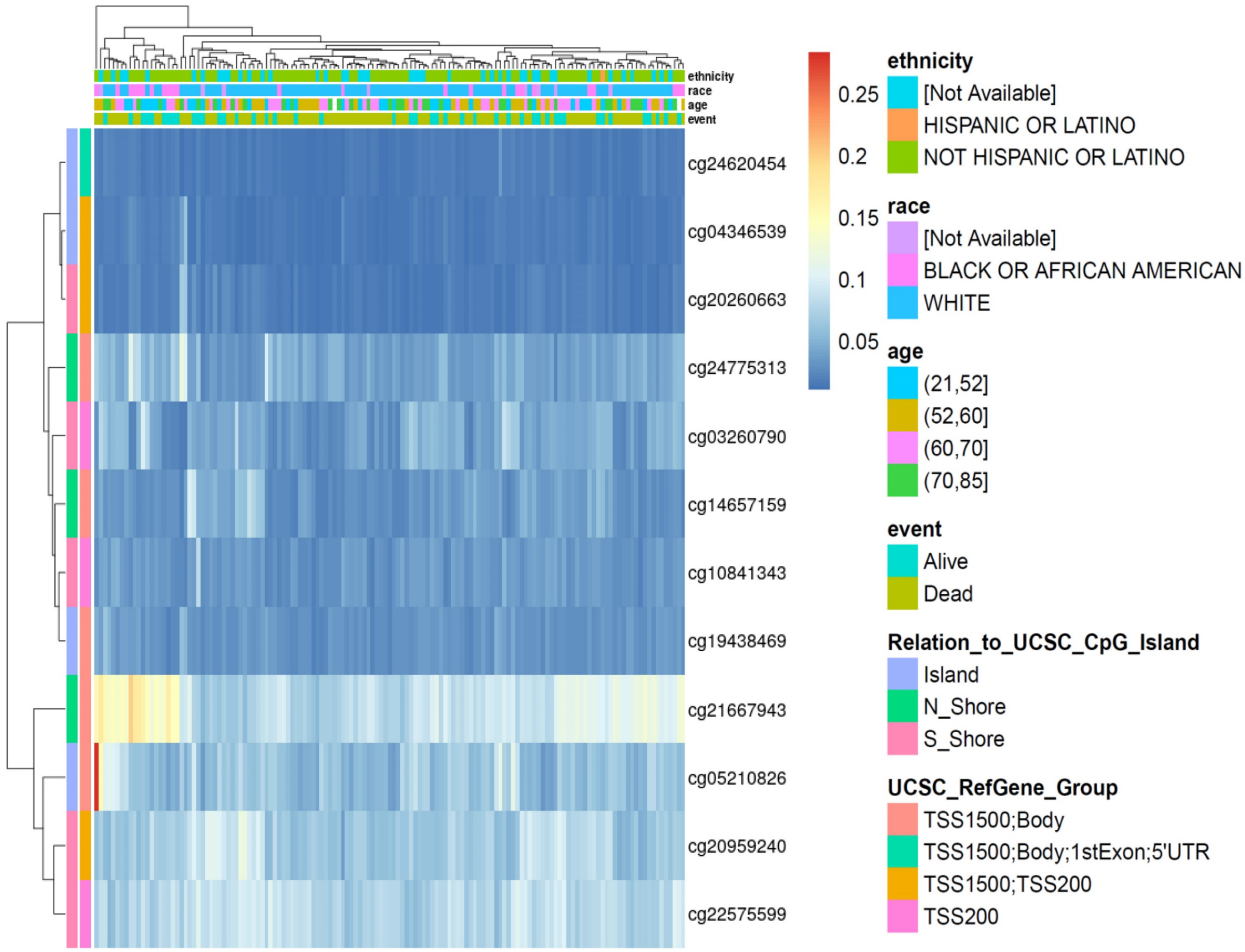
** DNA methylation heatmap of selected CpG sites associated with the ORMDL2 gene across brain tumor samples.** Rows represent individual CpG probes, while columns represent patient samples. The heatmap is annotated with clinical and demographic variables, including ethnicity, race, age group, and survival status (event). Methylation levels are color-coded from low (blue) to high (yellow). The legend to the right displays the categorical breakdowns for each annotation, including relation to CpG island regions and UCSC gene group classifications. Hierarchical clustering indicates variable methylation patterns across samples, suggesting potential epigenetic regulation of ORMDL2 in association with clinical features.

To understand the upstream regulation of ORMDL2, epigenetic alterations were explored using DNA methylation profiles from the TCGA 450K array. In total, 12 CpG probes within ±2 kb of the transcription start site (TSS) were extracted and visualized via unsupervised hierarchical clustering (Fig. 4). Probes such as cg21667943 and cg19438469, located within CpG islands overlapping the ORMDL2 promoter region (TSS200), exhibited consistent hypomethylation in patients with elevated ORMDL2 expression. In the methylation heatmap (Fig. 4), unsupervised clustering revealed a patient subset with concordant hypomethylation across multiple CpG sites, many of which overlapped with short survival and isocitrate dehydrogenase 1 (IDH1)-wild type tumors. This indicates that promoter hypomethylation may serve as an early activation mechanism for ORMDL2 in gliomagenesis. Stratification by clinical parameters revealed that hypomethylated CpG sites were predominantly found in patients who were older, deceased, or with an unmethylated methyl guanine methyl tranferase (MGMT) status. Data from ATAC-Seq in the TCGA Pan-Cancer cohort further showed increased chromatin accessibility surrounding the ORMDL2 promoter, especially in mesenchymal-like samples. Together, these data support a multi-layered regulatory model in which epigenetic derepression and chromatin remodeling synergistically drive ORMDL2 upregulation in GBM.

### ORMDL2 co-expression network reveals metabolic and mitochondrial dysregulation

**Figure 5:**
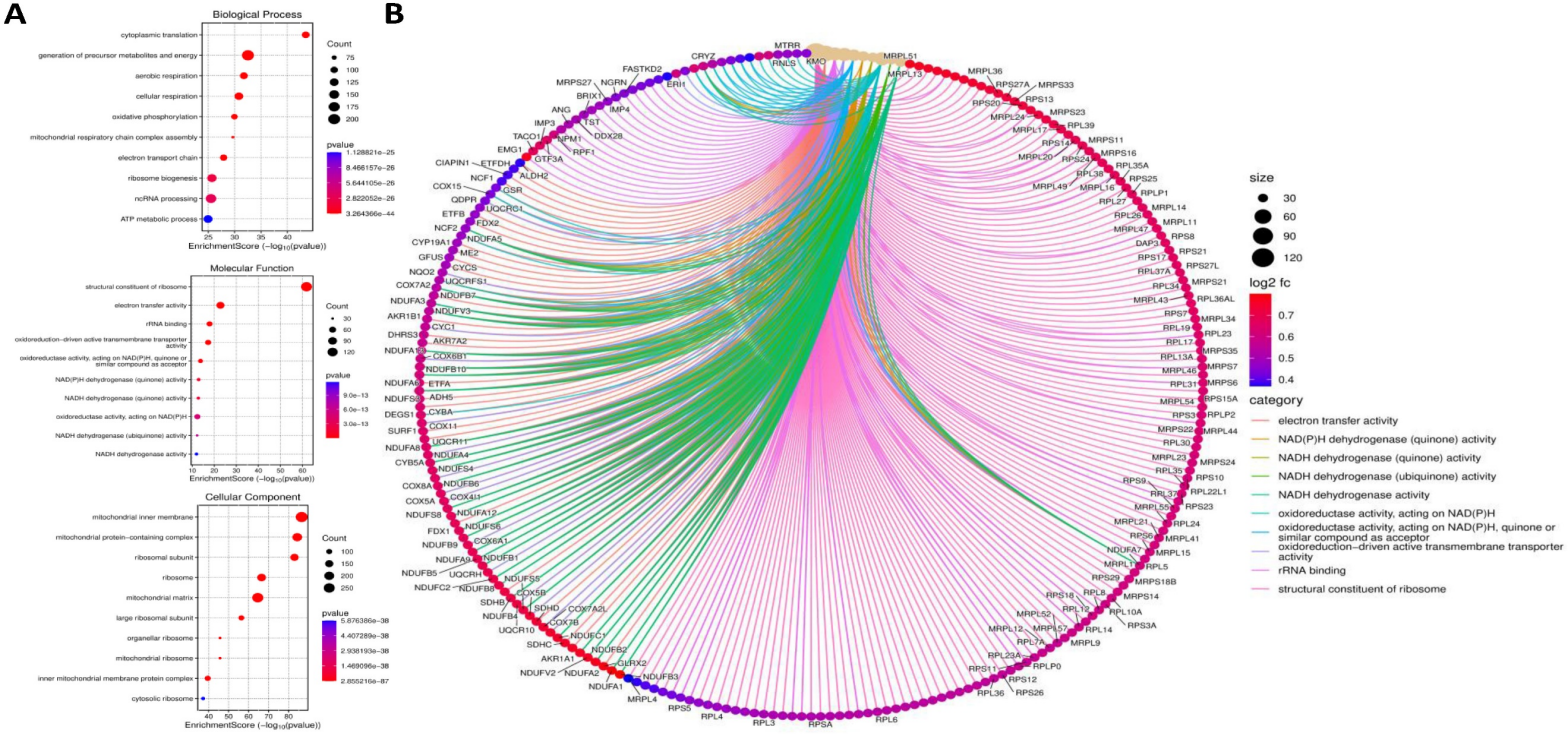
** Gene Ontology (GO) enrichment and functional interaction analysis of ORMDL2-associated differentially expressed genes.** (A) Dot plots representing the top enriched GO terms categorized by biological process (top), molecular function (middle), and cellular components (bottom). The x-axis denotes the enrichment score (-log₁₀ p-value), while the dot size represents the gene count, and color indicates statistical significance. Key biological processes include oxidative phosphorylation, mitochondrial respiratory chain complex assembly, and ATP metabolic processes. (B) Chord diagram visualizing functional interactions among significantly enriched genes and GO terms. Gene names are shown around the circle, with color-coded ribbons linking genes to their respective GO categories. Categories such as "electron transfer activity," "structural constituent of ribosome," and "NADH dehydrogenase activity" are highly represented. The ribbon color corresponds to the functional category, and node color intensity indicates log₂ fold change, with darker red indicating higher expression.

To dissect the functional role of ORMDL2, co-expressed genes were identified in the TCGA-GBM dataset using cBioPortal, and LinkedOmics, and Omics Playground v.3.4.1. The top 100 positively correlated genes (|Spearman *r*| ≥ 0.6, *p* < 0.05) were subjected to an enrichment analysis using clusterProfiler, Enrichr, and ShinyGO (Fig. 5). A Kyoto Encyclopedia of Genes and Genome (KEGG) analysis revealed that gene co-expressed with *ORMDL2* were enriched in pathways such as oxidative phosphorylation (OXPHOS), lysine degradation, non-alcoholic fatty liver disease, and neurodegenerative disease modules including Parkinson's and Huntington's disease, which were associated with mitochondrial dysfunction in GBM. Gene Ontology (GO) annotations indicated a strong association with mitochondrial compartments, including the proton-transporting ATP synthase complex, inner mitochondrial membrane, and respiratory chain complex I (Fig. 5). These genes were also involved in aerobic respiration, NADH dehydrogenase activity, and electron transfer, reinforcing the hypothesis that ORMDL2 is closely linked to bioenergetic flexibility and redox control. It was observed that several ORMDL2 co-expressed genes, such as NDUFA13, ATP5MC2, and COX7C, were components of the mitochondrial respiratory chain complex, indicating ORMDL2's likely involvement in aerobic glycolysis-OXPHOS transitions underlying metabolic plasticity in GBM. Furthermore, the co-enrichment of DDIT3 (CHOP) and ATF4 suggested a link to the UPR and ER stress buffering, placing ORMDL2 at the intersection of mitochondrial regulation and proteostasis adaptation.

### ORMDL2-associated genes mediate immune evasion and apoptotic resistance

**Figure 6:**
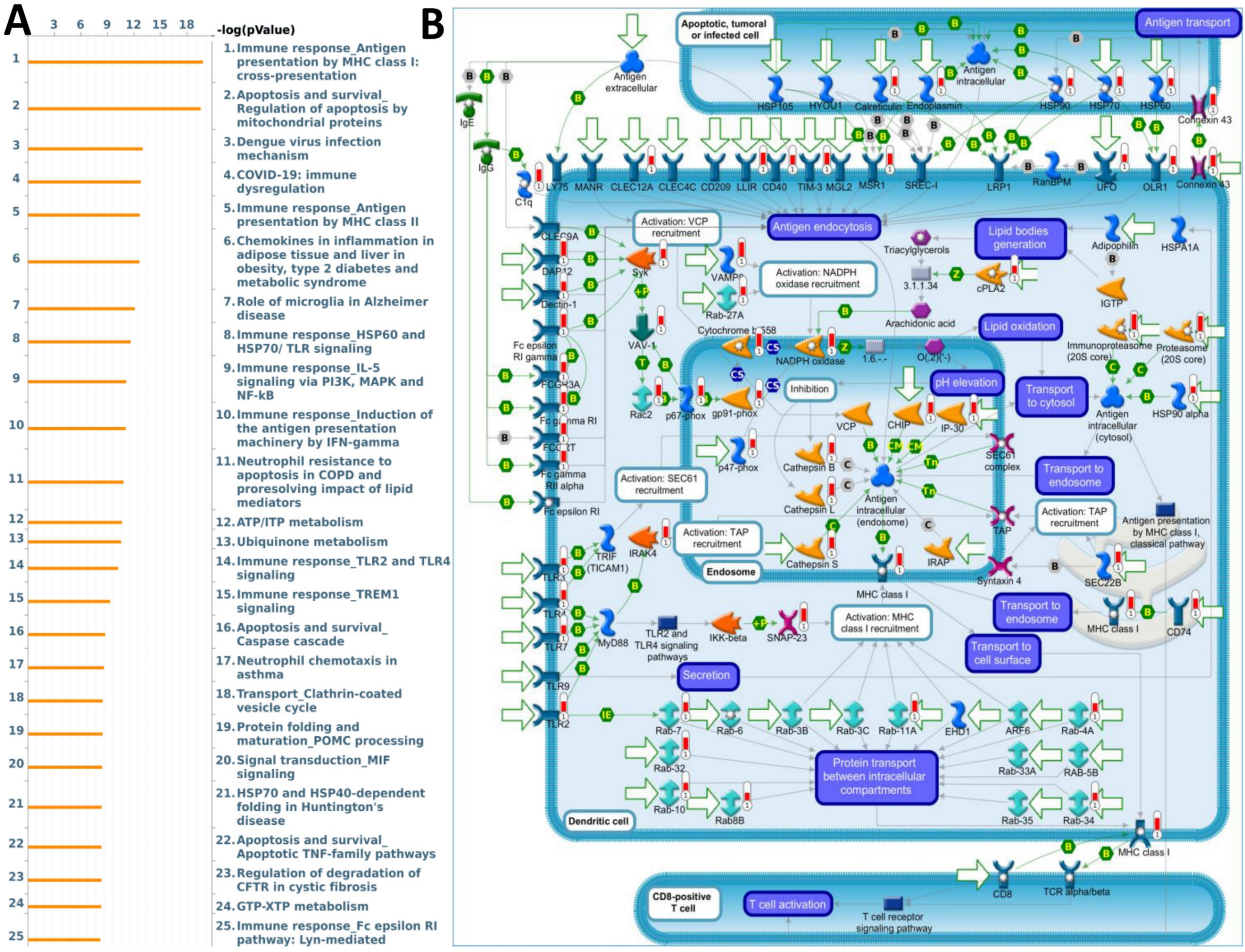
** MetaCore pathway enrichment analysis of ORMDL2 co-expression signature in GBM**. (A) The top 25 enriched pathways generated by MetaCore enrichment analysis using ORMDL2 co-expressed genes (Spearman *r* ≥ 0.6). Pathways are ranked by -log(*p*) values, with the most significant pathways involving antigen presentation via MHC class I cross-presentation, mitochondrial apoptosis, TREM1/TLR signaling, and the PI3K/IL-5/NF-κB axis, all of which play key roles in tumor immunity and immune evasion. (B) MetaCore process network map highlighting the MHC class I antigen presentation pathway. Key ORMDL2-associated genes are mapped to compartments including endosomes, transport vesicles, and cytosolic antigen-processing machinery, indicating ORMDL2's potential indirect role in modulating immune recognition of GBM cells. Specific involvement is noted in vesicle recruitment (Rab27a), proteasome processing (PSMB family), and ER-Golgi transport. These findings support the hypothesis that ORMDL2 regulates macromolecular immune networks in GBM, possibly influencing antigen processing and immune evasion.

The biological functions of ORMDL2 were further investigated using a co-expression network analysis and enrichment analysis based on MetaCore. The analysis revealed multiple key biological processes involving ORMDL2 co-expressed genes (Fig. 6A). Among these, the most significantly enriched pathway was “Immune response Antigen presentation by MHC class I: cross-presentation” (*p* = 1.55E-20), indicating a strong correlation between ORMDL2 and molecules essential for immune evasion. This pathway includes major immune-regulatory molecules such as cathepsin L/S/B, vesicle-associated membrane protein 8 (VAMP8), Rab guanosine triphosphatases (GTPases), Toll-like receptor 2/4/7 (TLR2/4/7), Fc gamma receptors, and heat-shock protein 90 (HSP90) family chaperones, many of which participate in vesicle fusion, lysosomal trafficking, and impaired antigen presentation under tumor progression (Fig. 6B; Supplementary Table 2, Rank #1). This is particularly relevant given that evasion of immune surveillance is a hallmark of GBM progression. Disruption of antigen cross-presentation via downregulation or misrouting of MHC class I complexes may facilitate ORMDL2-expressing GBM cells to evade cytotoxic T cell detection. Furthermore, the involvement of calreticulin, ER chaperones (HSP90, HSP70, endoplasmin), and TLRs suggests that ORMDL2 may mediate broader effects on ER stress signaling and immunoproteasome dysfunction. In addition to immune regulation, ORMDL2 co-expressed genes were highly enriched in the “Apoptosis and survival; Regulation of apoptosis by mitochondrial proteins” pathway (Supplementary Data S1; Supplementary Table 2, Rank #2), including BAX, Bak, cytochrome c, voltage-dependent anion channel ½ (VDAC1/2), second mitochondrion-derived activator of caspase (SMAC)/Diablo, and multiple caspases. These components regulate mitochondrial membrane permeabilization and intrinsic apoptotic signaling, implying that ORMDL2 may suppress apoptosis under chemotherapeutic or hypoxic stress by modulating mitochondrial stability. Interestingly, several of the enriched pathways, such as TLR2/4 signaling, Fc receptor signaling, and immune dysregulation during COVID-19, shared overlapping immune-suppressive or inflammatory traits, suggesting that ORMDL2 may be a broad coordinator of tumor inflammation, immune evasion, and apoptosis resistance. To further validate these observations, we examined the normalized enrichment scores of ORMDL2 high versus low groups using a GSEA. Consistent with MetaCore, the GSEA identified positive enrichment of immune-related pathways such as the interferon-gamma response, antigen presentation, macrophage activation, and ER stress-associated unfolded protein response (UPR) in the ORMDL2-high group. Collectively, these enrichment analyses suggested that ORMDL2 may function at the intersection of immune presentation machinery suppression and mitochondrial anti-apoptotic buffering, contributing to GBM's resilience to immune clearance and stress-induced cell death.

### Single-cell transcriptomics reveal ORMDL2 enrichment in stroma cells

**Figure 7:**
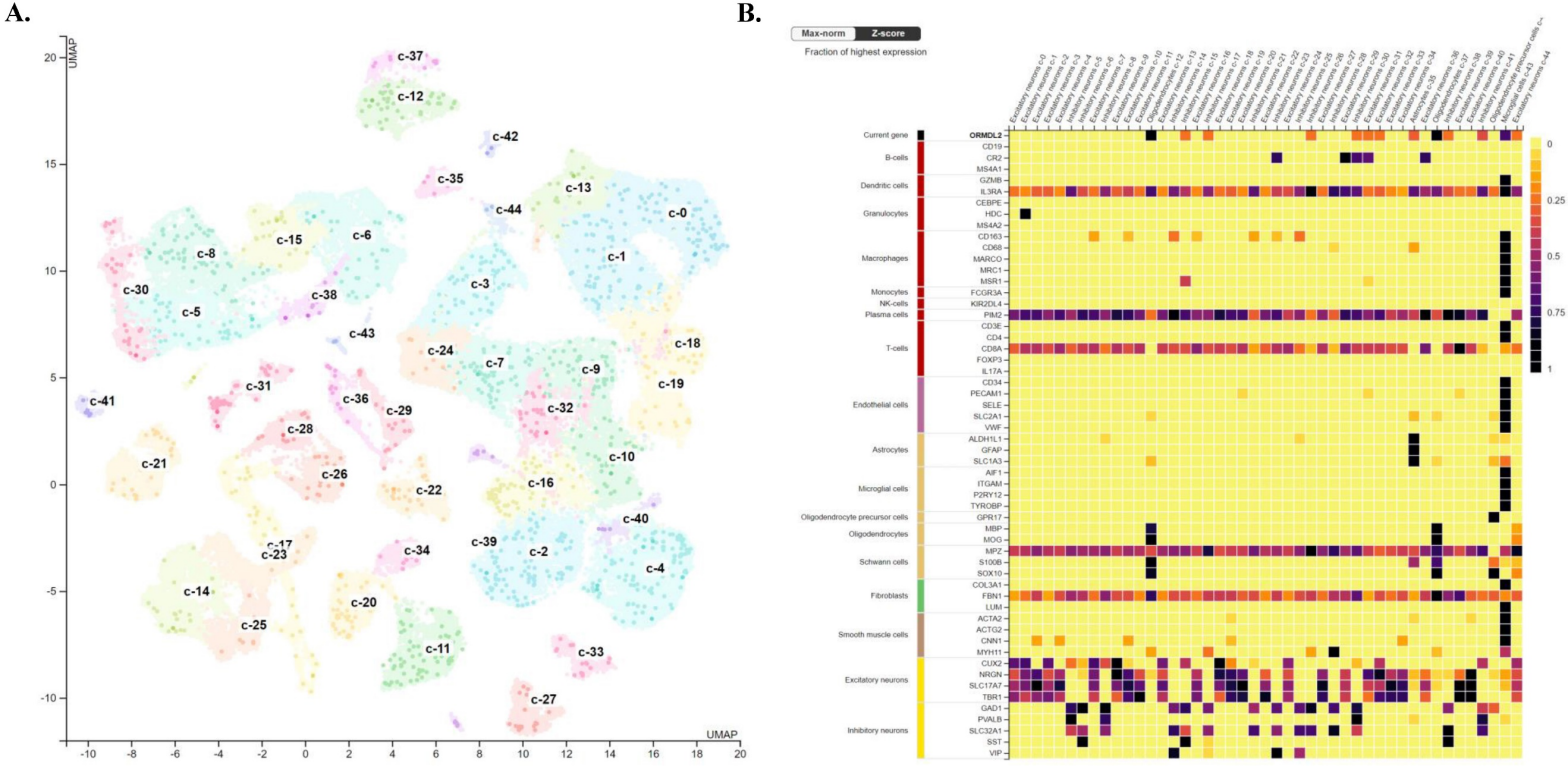
** Analysis of ORMDL2 in tumor microenvironment characteristics in brain.** (A) UMAP projection of single-cell RNA sequencing data illustrating clustering of brain cells into distinct cell-type populations. Each cluster is color-coded and annotated with its corresponding cell type, including excitatory neurons, inhibitory neurons, astrocytes, oligodendrocytes, microglia, and others, as indicated in the legend on the left. Expression levels of ORMDL2 (in nTPM) are overlaid, highlighting its distribution across different clusters. (B) Heatmap depicting the normalized expression (Max-norm Z-score) of ORMDL2 (top row) alongside canonical marker genes for various immune and stromal cell types from the Human Protein Atlas (HPA). Columns represent the defined clusters from panel A, and rows correspond to gene markers, with associated cell types annotated on the left. Color coding on the left group's markers by major cell type categories with shared functional properties. This analysis reveals the cellular specificity and relative enrichment of ORMDL2 expression within the brain microenvironment.

To further investigate the cellular context and microenvironmental distribution of ORMDL2 in GBM, publicly available scRNA-Seq datasets were analyzed from the Human Protein Atlas (HPA) database and CancerSCEM, focusing on GBM tissues. The analysis provided insights into the expression pattern of ORMDL2 across transcriptionally distinct cell types within the tumor microenvironment (TME). As visualized in the UMAP plot (Fig. 7A, top), individual cells were clustered based on their transcriptional profiles and annotated according to known functional cell types, including immune cells (such as microglia and macrophages), glial cells (including astrocytes and oligodendrocytes), endothelial cells, and neuronal populations. Each dot represents a single cell, and the intensity of ORMDL2 expression was depicted by a color gradient scaled to the percentage of maximal normalized transcript expression per million (log₂[nTPM+1] / log₂[max(nTPM)+1] × 100). The accompanying bar chart (Fig. 7A) shows cumulative ORMDL2 transcript levels across the cell-type-defined clusters, confirming that immune-related and glial populations exhibited higher average nTPM expression levels of ORMDL2. To further refine the cell-type specificity, a heatmap was generated to compare ORMDL2 expression with canonical marker genes across all single-cell clusters (Fig. 7B). Z-score normalization was applied to facilitate comparisons across genes and clusters. The results demonstrated that ORMDL2 expression was enriched in cell populations associated with microglia/macrophages, astrocytes, and oligodendrocytes, while neuronal and endothelial lineages showed relatively lower expression levels. These observations suggest a functional association of ORMDL2 with glial immune cells and tumor-supportive stromal components.

The enrichment of ORMDL2 in glial and myeloid lineages was consistent with its proposed roles in ER stress regulation, lipid metabolism, and immunosuppressive signaling. Microglial populations with high ORMDL2 expression also co-expressed immunoregulatory markers such as cluster of differentiation 163 (CD163), trigerring receptor expressed on myeloid cells 2 (TREM2), and macrophages mannose receptor 1 (MRC1), implicating *ORMDL2* in the maintenance of M2-like macrophage polarization within the GBM microenvironment. Given the known role of ORMDL proteins in sphingolipid biosynthesis and ER homeostasis, ORMDL2 may contribute to cellular adaptation under hypoxic or inflammatory stress, which is a niche characteristic of the TME. Additionally, prior studies have shown that ER stress-related genes modulate antigen presentation, phagocytosis, and cytokine secretion in myeloid-derived suppressor cells (MDSCs) and TAMs. The specific expression of ORMDL2 in these immunosuppressive populations supports its candidacy as a modulator of tumor-associated immune evasion. These scRNA-Seq analysis provided evidence that ORMDL2 was not ubiquitously expressed but rather selectively enriched in stromal and immune cell subtypes within GBM. These findings underscore its potential as a lineage-specific therapeutic target, particularly in modulating macrophage-driven immune suppression and ER stress adaptation in the TME.

### High ORMDL2 expression correlates with drug resistance and predicts TMZ sensitivity

**Figure 8:**
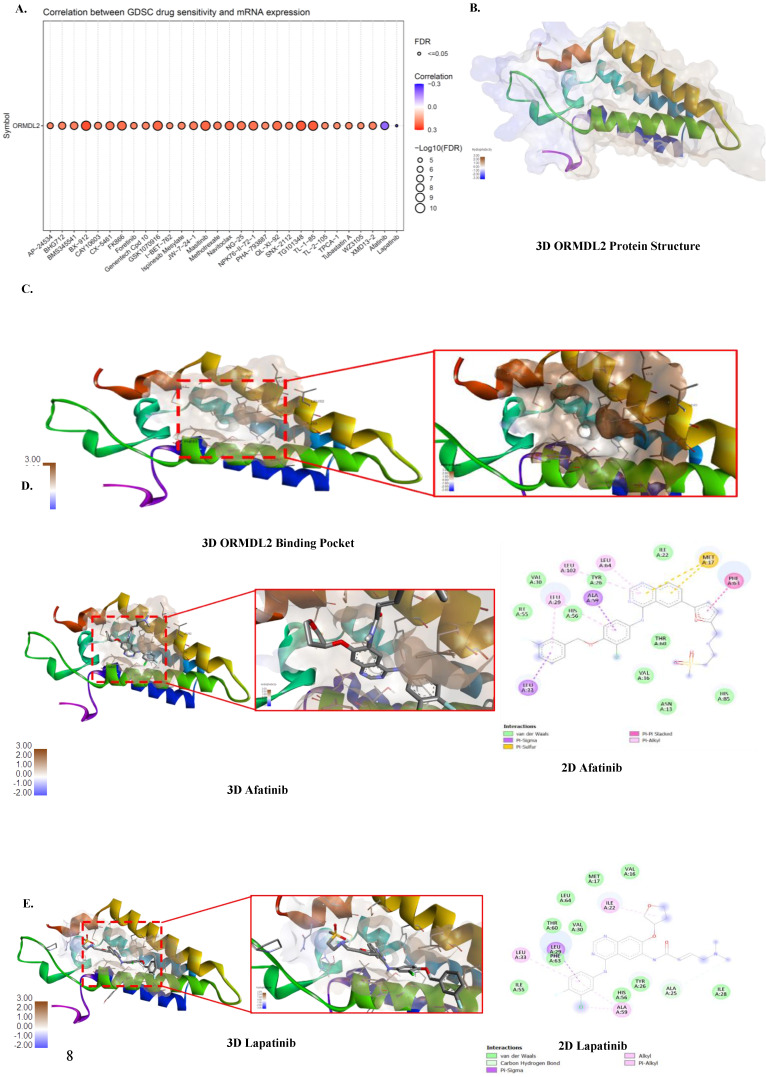

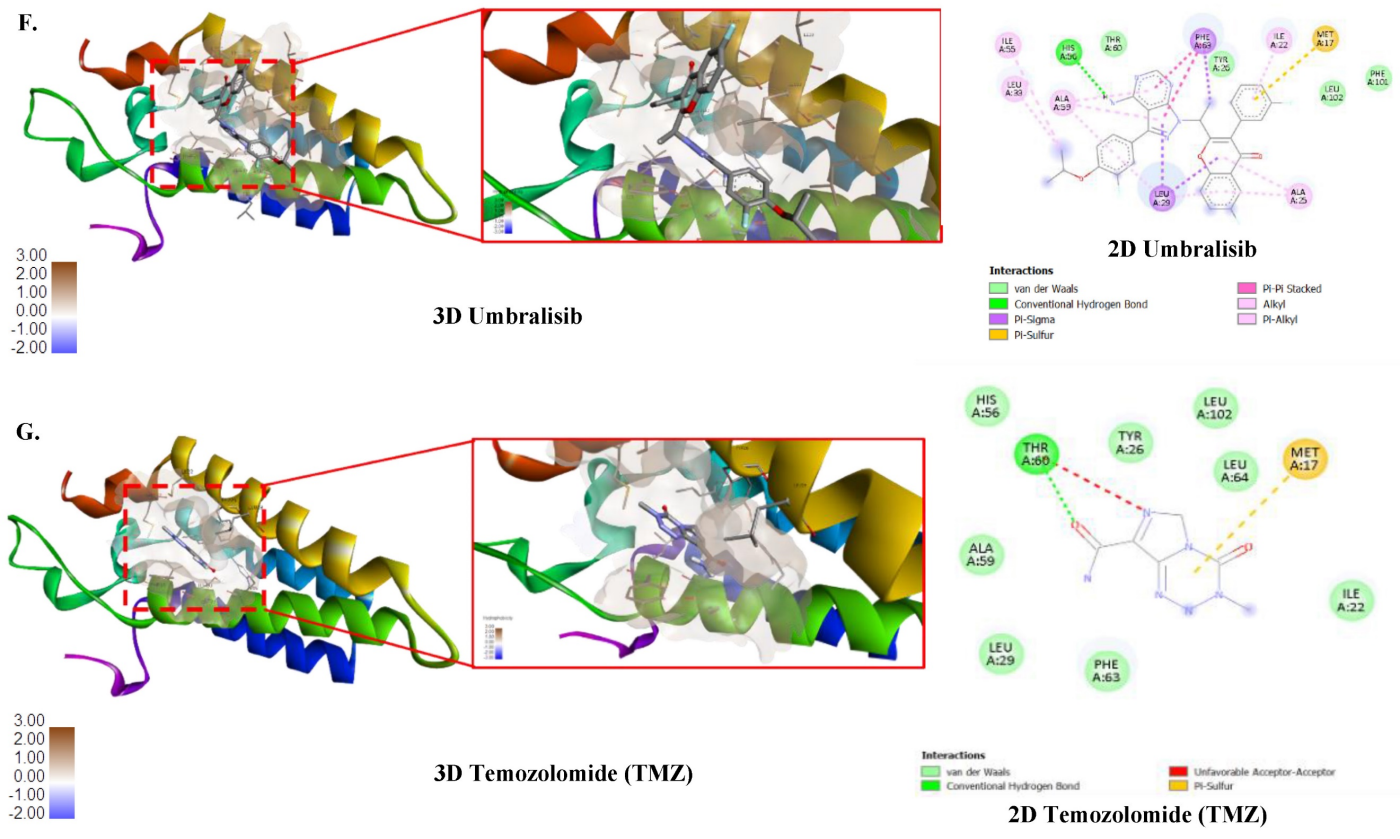
** Correlation of drug sensitivity with ORMDL2 and molecular docking analysis.** (A) Bubble plot showing the correlation between ORMDL2 gene expression and the sensitivity of cancer cell lines to various drugs from the Genomics of Drug Sensitivity in Cancer (GDSC) database. The color of each bubble indicates the Pearson correlation coefficient between ORMDL2 expression and drug response (red: positive, blue: negative), while the size of each bubble represents the statistical significance (-log₁₀ false detection rate (FDR)). Only drugs with significant associations (FDR ≤ 0.05) are shown. (B) 3D structure of the ORMDL2 protein as predicted by AlphaFold. (C) Binding Pocket of the ORMDL2 protein. (D-G) 3D and 2D binding pocket of ORMDL2 with Afatinib (-7.6 Kcal/Mol), Lapatinib (-8.0 Kcal/Mol), Umbralisib (-8.6 Kcal/Mol), and TMZ (-5.5 Kcal/Mol).

To assess the therapeutic implications of ORMDL2 dysregulation in GBM, we conducted pharmacogenomic analyses was conducted using the Genomics of Drug Sensitivity in Cancer (GDSC) and Cancer Therapeutics Response Portal (CTRP) datasets. Correlation analyses were performed to identify drugs whose sensitivity (measured by 50% inhibitory concentration (IC_50_ values) were significantly associated with ORMDL2 mRNA expression levels across GBM cell lines. In the GDSC dataset (Fig. 8A), ORMDL2 expression was positively correlated with reduced sensitivity to a broad spectrum of compounds, including DNA replication inhibitors (Gemcitabine, Etoposide, Mitoxantrone), protein kinase inhibitors (BX-912, AZD7762, Foretinib, WZ-3146), and metabolic inhibitors (Methotrexate, Bortezomib). These drugs share mechanisms related to DNA damage response, cell cycle arrest, and oxidative stress induction. Among these, strong correlations were observed with Foretinib and CX-5461, agents targeting PI3K/mammalian target of rapamycin (mTOR) and RNA polymerase I, suggesting that ORMDL2 may modulate broader macromolecular stress signaling. These results reinforce the hypothesis that ORMDL2-expressing cells exhibit adaptive resistance not only to genotoxic compounds but also to inhibitors of protein quality control and stress signaling pathways. These findings highlight ORMDL2 as a predictive marker for drug resistance across diverse mechanistic classes, implicating it in cellular programs that buffer against DNA damage, ER stress, and apoptosis, central vulnerabilities in tumor therapy.

### Molecular docking predicts druggable interactions at ORMDL2's ceramide-regulatory domain

Binding affinity of several drugs was tested to check their efficacy against ORMDL2 (Fig. 8B-G). The best binding affinity was found for the HDAC inhibitor umbralisib with a binding affinity of (-8.6 Kcal/Mol). This was followed by alpelisib, an HDAC inhibitor, also lapatinib from GDSC, an Epidermal Growth Factor Receptor (EGFR) inhibitor and Human Epidermal Growth Factor Receptor 2 (HER2). TMZ an alkylating agent that exerts its anti-cancer effect by damaging DNA was used as the control. However its binding affinity was low (-5.5 Kcal/Mol). Results of molecular docking produced the predicted binding affinities of *ORMDL2* with Afatinib and Lapatinib. Both binding scores for afatinib and lapatinib, at -7.6 kcal/mol and -8.5 kcal/mol respectively, revealed good binding affinities. Therefore, this indicated that lapatinib was more favorable compared to afatinib.

### *In vitro* study of ORMDL2 expression across GBM cell lines

**Figure 9:**
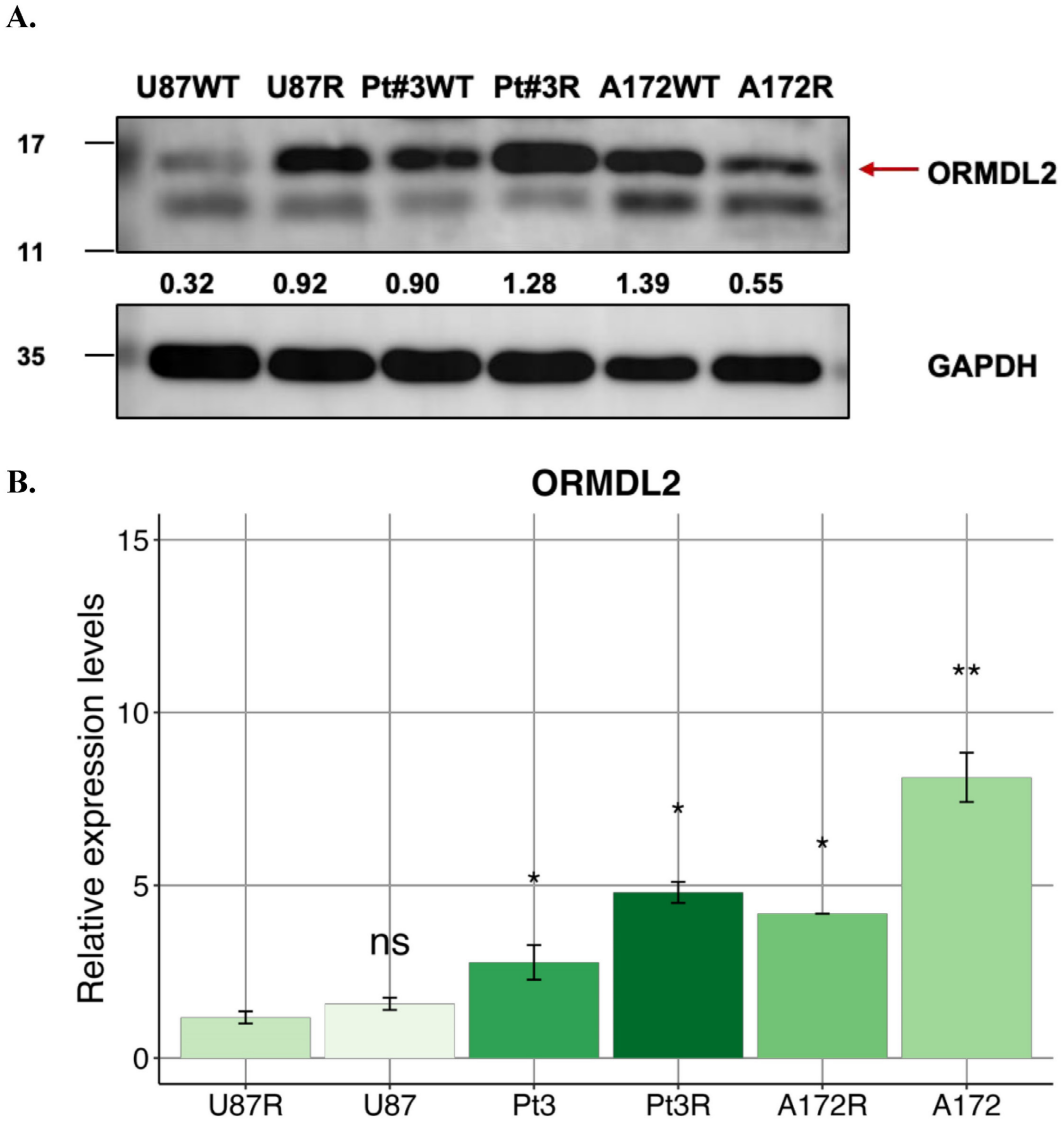
** ORMDL2 basal level expression in glioblastoma multiforme (GBM) cell lines.** (A) Western blot analysis performed on all GBM cell lines to determine the basal level of ORMDL2 expression in those cell lines. (B) A RT-qPCR analysis was performed on A172, U87-MG, and Pt#3 patient-derived GBM cells to determine baseline ORMDL2 expression. A172 cells demonstrated the highest ORMDL2 expression. β-actin was used as an internal control. Data are represented as the mean ± SD from three biological replicates (*p* < 0.01).

To validate our bioinformatics findings, Western blotting (Fig. 9A) and RT-qPCR (Fig. 9B) were applied to evaluate ORMDL2 expression across different GBM cell lines. Among the tested lines, the patient-derived Pt#3R cells exhibited the highest protein expression, followed by the A172 cell line. U87R cells also showed higher expression compared to their parental U87WT cells. At the mRNA level, RT-qPCR results indicated that A172 had the strongest ORMDL2 expression among the parental cell lines (U87 and Pt#3), while both U87 cell types showed low expression of the target gene.

## Discussion

GBM remains one of the most lethal malignancies, marked by rapid progression, profound intra-tumoral heterogeneity, and resistance to multimodal therapy. While most of research has centered on transcriptional drivers, epigenetic modulators, and tumor-intrinsic oncogenic pathways, increasing evidence suggests that ER-resident proteins and immune metabolic regulators are equally pivotal in shaping the tumor phenotype. In this context, this study identified ORMDL2 (orosomucoid-like protein 2) as a novel immune-metabolic macromolecule that may function as a regulatory hub of GBM progression and therapeutic evasion. ORMDL2 belongs to a highly conserved family of ER membrane-bound macromolecules involved in sphingolipid biosynthesis, calcium signaling, and ER stress modulation 74, 75.

Historically, its functions were largely extrapolated from studies in inflammatory diseases, such as asthma and metabolic syndrome, where ORMDL proteins were shown to regulate ceramide production and the UPR. However, the functional role of ORMDL2 in the context of human malignancies, particularly brain tumors still has remained elusive. Our study provides the first comprehensive multi-omics characterization of ORMDL2 in GBM, demonstrating its elevated expression, adverse prognostic implications, and association with immune evasion and apoptosis resistance pathways. Through pan-cancer transcriptomic analysis, ORMDL2 was shown to be overexpressed in brain tumor tissues relative to normal brain tissues, with its expression correlating significantly with clinical features such as IDH mutation status, MGMT promoter methylation, and World Health Organization (WHO) grade. More importantly, higher ORMDL2 expression conferred poorer overall survival in three independent cohorts (of TCGA, and CGGA), even after adjusting for age group and therapy. These observations suggest that ORMDL2 is not merely a passive marker but may actively contribute to disease progression.

A deeper analysis of co-expressed gene networks and pathway enrichment revealed that ORMDL2 was embedded in functional modules governing antigen processing and presentation, apoptosis regulation via mitochondrial proteins, and innate immune signaling, particularly via TLRs and MHC molecules. Remarkably, MetaCore enrichment highlighted “Antigen presentation by MHC class I cross-presentation” as the top-ranked pathway, implicating ORMDL2 in dendritic cell-mediated cross-priming and tumor antigen evasion. This is in line with recent findings that ER stress and altered lipid metabolism in tumor cells can suppress antigen presentation, leading to ineffective immune surveillance. Our results further aligned with the data from The Cancer Immunome Atlas (TCIA) and TISIDB, which showed a strong co-expression of ORMDL2 with immune checkpoint genes including CD274 (PD-L1), HAVCR2 (TIM-3), and CD276 (B7-H3). These findings suggest that ORMDL2 may shape an immunosuppressive tumor microenvironment (TME) via regulating MHC-I/II expression and modulation of dendritic cell and macrophage phenotypes. The scRN-Seq analysis from the HPA revealed that ORMDL2 was highly expressed in TAMs and glioma stem-like cells (GSCs), two major contributors to immune evasion and therapeutic resistance in GBM. Notably, TAMs with high ORMDL2 expression co-expressed M2-polarization markers (CD163 and MRC1) and ceramide metabolism regulators, indicating a potential role in lipid-reprogrammed immunosuppression. GSCs, on the other hand, exhibited a robust ORMDL2 signature along with elevated ER stress markers, raising the possibility that ORMDL2 may aid in the maintenance of stemness and chemoresistance through ER homeostasis. This is particularly relevant as both TAMs and GSCs are known to support tumor recurrence after TMZ therapy, often by secreting cytokines that remodel the extracellular matrix or inhibit T cell infiltration.

It was notable that pharmacogenomic profiling via the GDSC and CTRP databases did not show a strong direct correlation between ORMDL2 expression and TMZ sensitivity. This finding suggests that ORMDL2 might not act through canonical alkylating resistance pathways such as MGMT upregulation, but rather influences broader macromolecular mechanisms, particularly ER stress buffering, metabolic reprogramming, and immune modulation, that collectively confer drug tolerance. Interestingly, ORMDL2 expression was positively associated with resistance to agents targeting the DNA damage response (DDR), including ATR and ATM inhibitors, and oxidative stress regulators such as PARP inhibitors. These associations imply that ORMDL2-expressing cells may harbor enhanced survival capacity under genotoxic stress, potentially via maintenance of sphingolipid homeostasis, ER stress tolerance, and mitochondrial regulation^76^. Furthermore, our molecular docking simulation using AutoDock Vina identified two small molecules with high binding affinities for ORMDL2: afatinib and lafatinib. This finding aligns with previous studies showing that targeting ceramide metabolism can enhance immunogenic cell death and restore T cell function in GBM. Thus, ORMDL2 represents a potentially druggable macromolecular target that intersects with both metabolic vulnerability and immune regulation in GBM. From a structural biology perspective, ORMDL2's coiled-coil domains and transmembrane topology support its capability to scaffold protein complexes at the ER membrane. Previous studies have implicated ORMDL2 in the formation of multi-protein complexes with serine palmitoyltransferase (SPT), ER chaperones (HSP90B1), and calcium-binding proteins, which collectively modulate the ER stress response and sphingolipid flux 77. Given our co-expression network data highlighting links to calreticulin, HSP70, and MHC-I processing components, it is plausible that ORMDL2 functions as an ER-resident organizer of antigen processing hubs, although this hypothesis requires further biochemical validation.

## Conclusions

In conclusion, our integrative analysis positions ORMDL2 as a multi-functional macromolecule that orchestrates ER stress, immune evasion, and chemoresistance in GBM. Unlike canonical oncogenes or immune checkpoints, ORMDL2 operates at the interface of lipid metabolism, proteostasis, and immunoregulation, offering a unique and druggable node within the GBM molecular network. Targeting ORMDL2 may open new therapeutic avenues that combine metabolic reprogramming with immune restoration, strategies that are urgently needed to overcome resistance in GBM.

## Supplementary Material

Supplementary figures and tables.

## Figures and Tables

**Figure 10:**
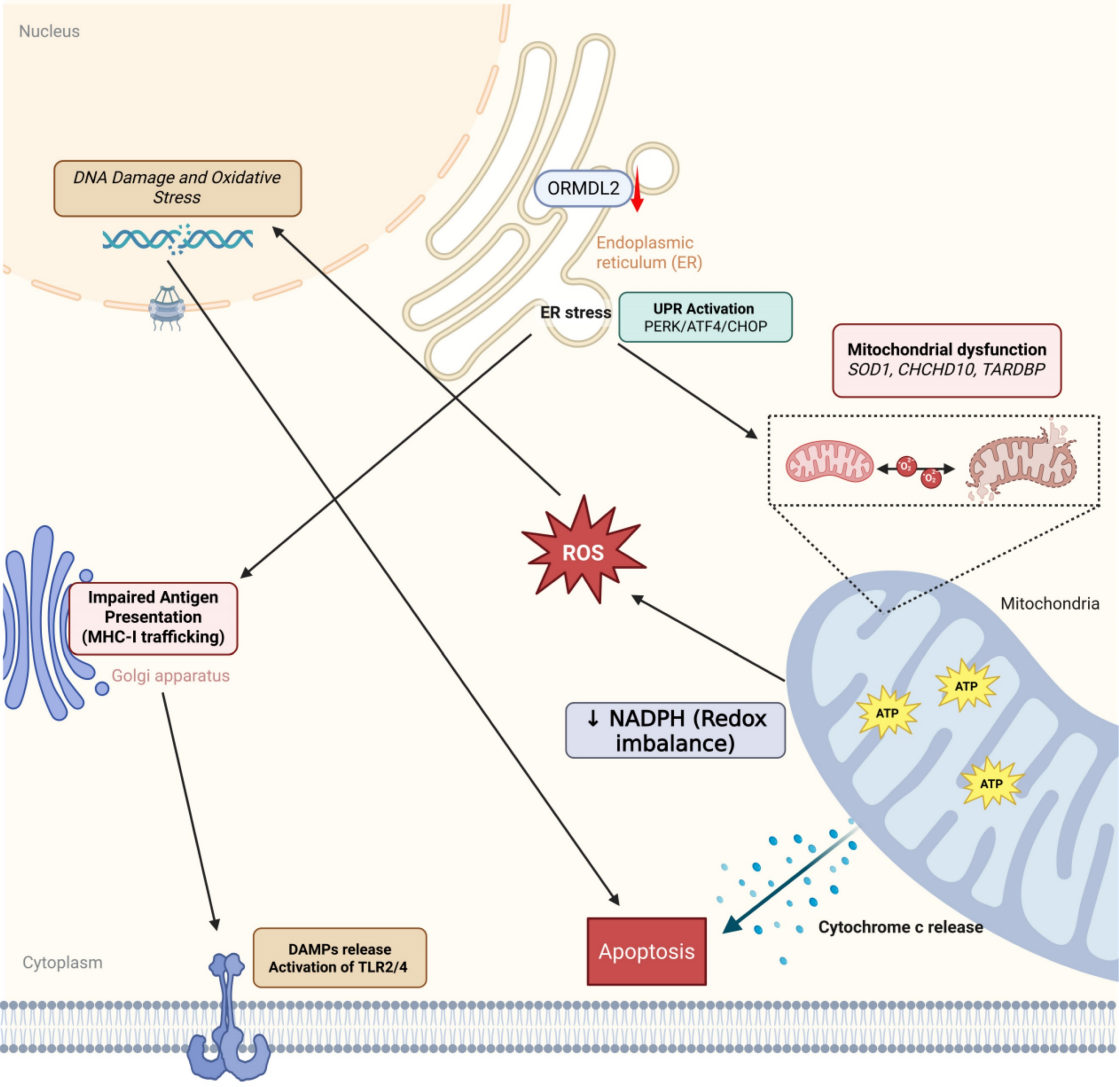
** ORMDL2-mediated immune-metabolic stress in glioblastoma.** Bioinformatics analysis suggests that ORMDL2 may participate in the regulation of ER stress responses, including the UPR including PERK, ATF4, CHOP signaling. This dysregulation is potentially linked to impaired mitochondrial function, redox imbalance, and increased reactive oxygen species (ROS), which in turn are associated with DNA damage and apoptotic signaling. Furthermore, altered ORMDL2 expression may influence MHC-I antigen presentation pathways, thereby modulating immune recognition. The release of damage-associated molecular patterns (DAMPs) under stress conditions could engage TLR2/4-related immune signaling. Collectively, these observations suggest that ORMDL2 may serve as a regulatory node connecting metabolic stress and immune evasion in glioblastoma.
