# Supplementary material for: Targeting ORMDL2 in glioblastoma through integrated landscape of high-throughput sequencing and pharmacogenomic analysis
Source: Int J Med Sci. 2025 Sep 27;22(15):4102–18. doi: 10.7150/ijms.116954 (PMC12492375; doi:10.7150/ijms.116954)
Supplement: Supplementary file 1 — Supplementary figures and tables. [file ijmsv22p4102s1.pdf]

Supplementary Data

A

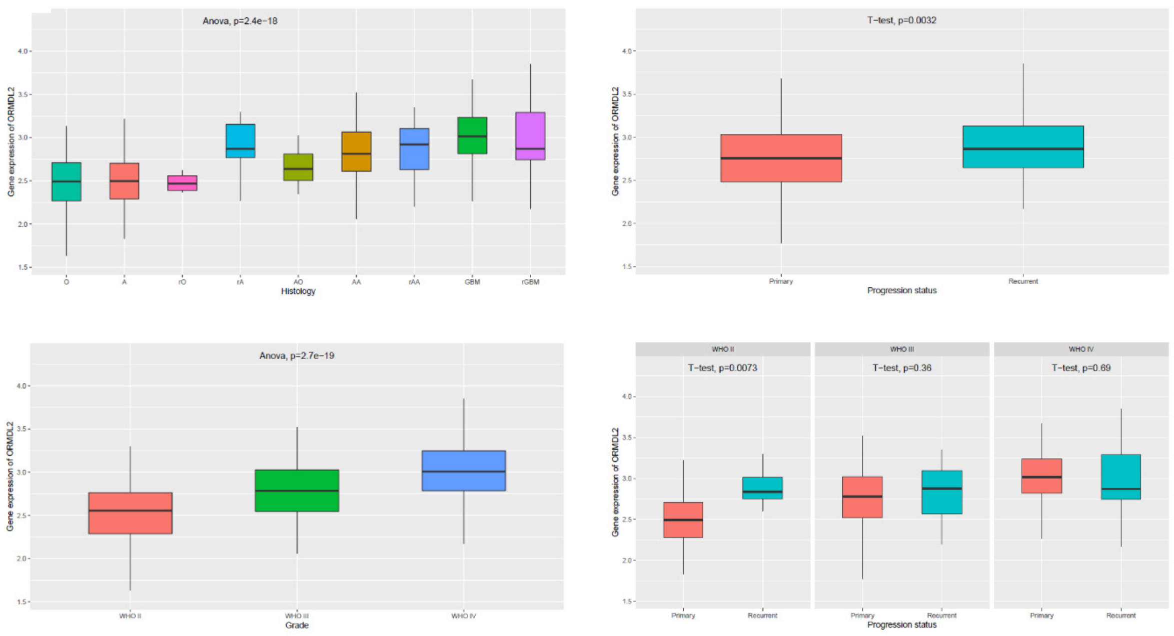

Supplementary Data S1: (A) Boxplots showing ORMDL2 mRNA expression in Chinese Glioma Genome Atlas (CGGA) datasets stratified by clinicopathological features. Top left: Differential expression of ORMDL2 among glioma histological subtypes (astrocytoma, oligodendroglioma, oligoastrocytoma, glioblastoma), p < 2.4e–18 by ANOVA. Top right: Comparison of ORMDL2 levels between primary and recurrent gliomas (p = 0.0032, t-test). Bottom left: Significant upregulation of ORMDL2 with increasing WHO tumor grade (p < 2.7e–19). Bottom right: Stratified analysis in WHO II–IV gliomas shows elevated ORMDL2 in recurrent tumors (WHO II: p = 0.0073; WHO III: ns; WHO IV: ns).

A.

B.

C.

D.

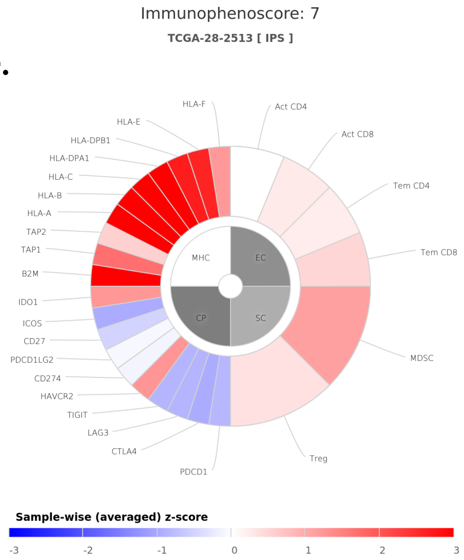

Supplementary Data S2: (A) Kaplan–Meier survival (KM Plotter) curves stratified by ORMDL2 expression levels across multiple subgroups in the CGGA dataset. High ORMDL2 expression is significantly associated with shorter overall survival ($p < 0.0001$). (B) Box plot showing elevated ORMDL2 expression in CGGA samples based on clinical patients' status. (C) GO enrichment dot plot illustrating biological processes associated with ORMDL2 in CGGA. (D) Comparison of ORMDL2 expression levels relative to Immunophenoscore (IPS), highlighting immune-related associations.

A. Correlation ORMDL2 with EGFR Family

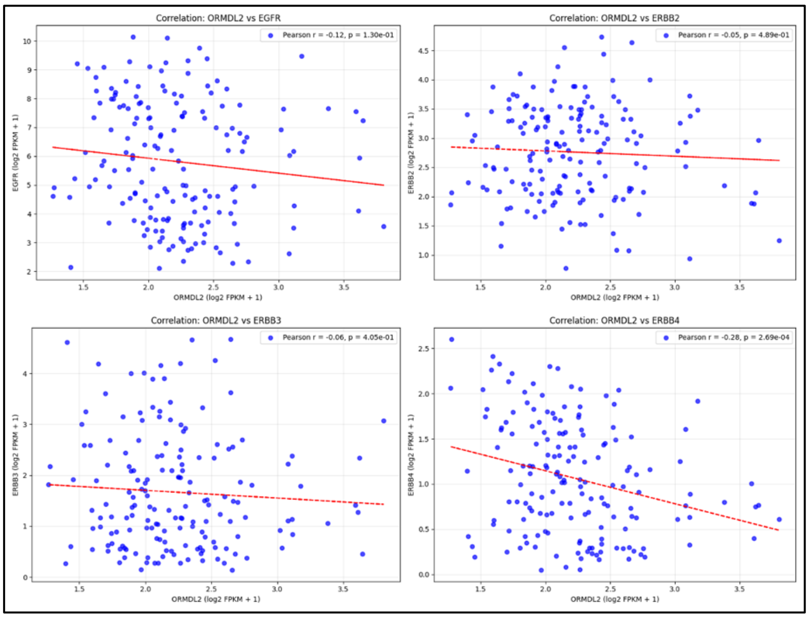

B. Correlation ORMDL2 with Cell Proliferation Marker

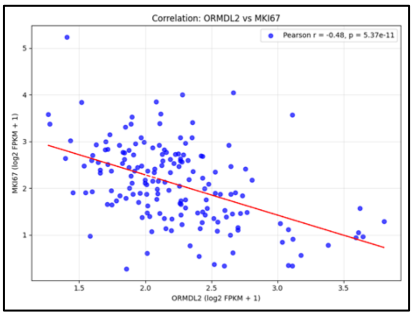

C. Correlation ORMDL2 with Apoptosis Regulator

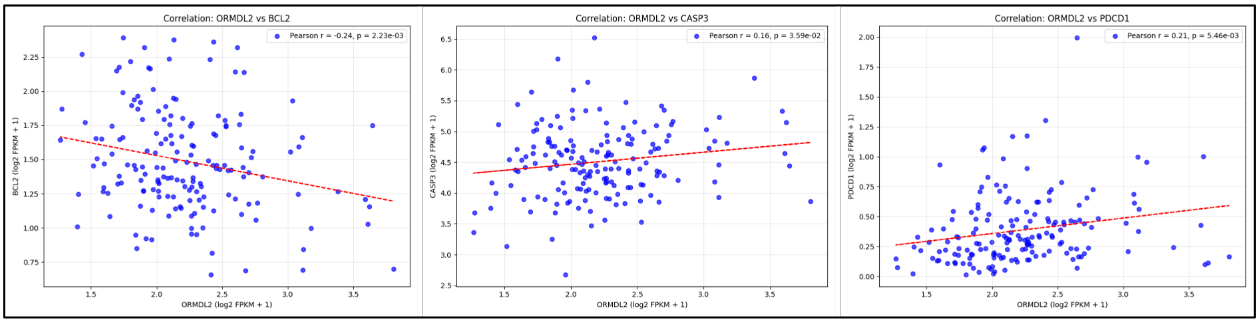

D. Correlation ORMDL2 with Immune Checkpoint

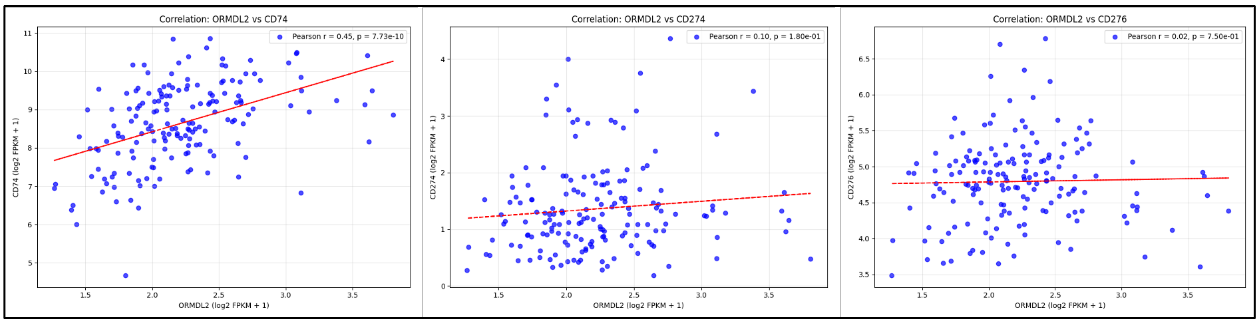

Supplementary Data S3: List of correlated markers against ORMDL2 expression. (A). Correlation ORMDL2 with EGFR Family (EGFR; ERBB2; ERBB3; ERBB4). (B) Correlation of ORMDL2 with Cell Proliferation Marker (MKI67). (C) Correlation ORMDL2 with Apoptosis Regulator (BCL2; Caspase-3). (D) Correlation ORMDL2 with Immune Checkpoint (CD74; CD274 [PD-L1]; CD276). The expression of ORMDL2 is located on the X axis while the Y axis contains the expression of the markers.

A.

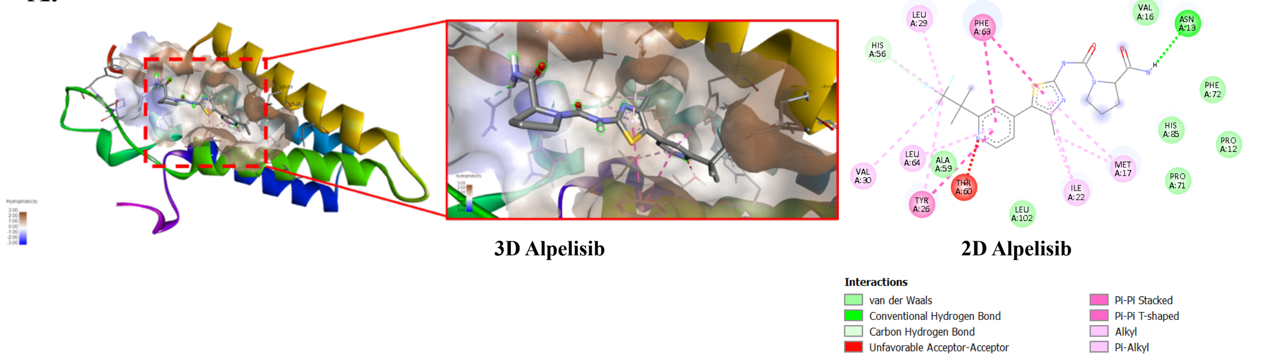

3D Alpelisib

2D Alpelisib

B.

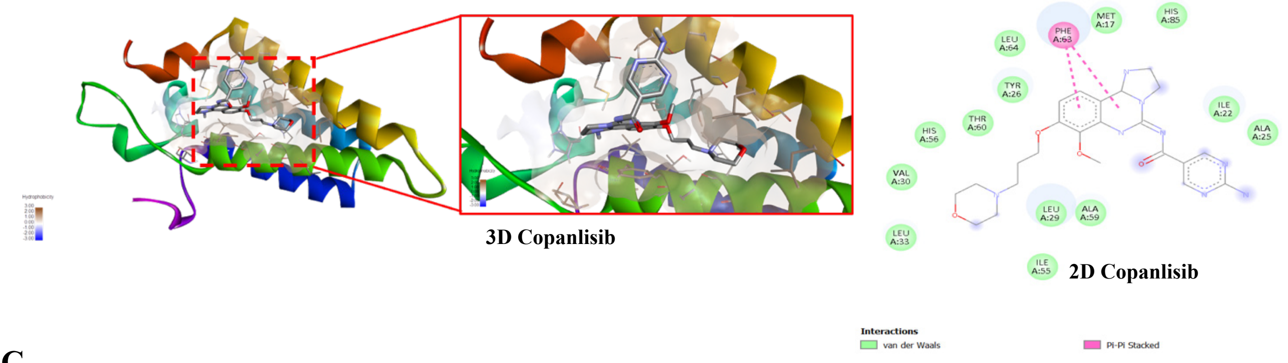

3D Copanlisib

2D Copanlisib

C.

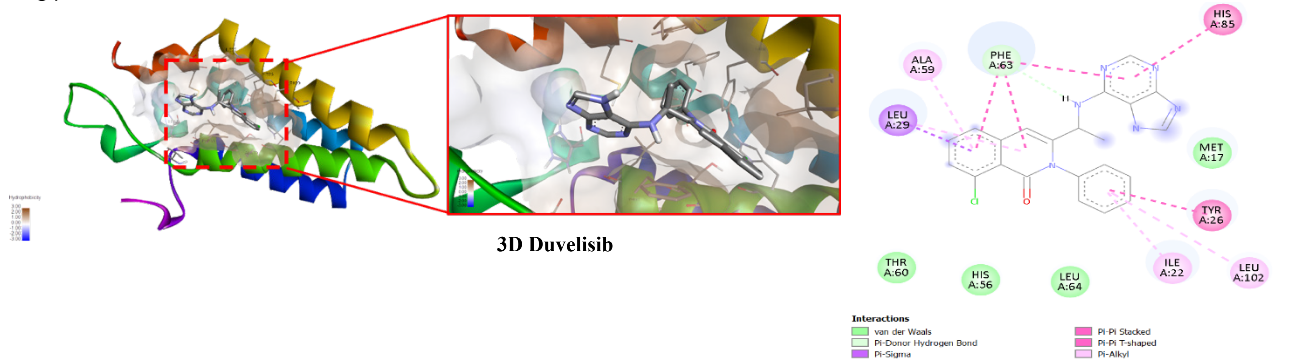

3D Duvelisib

2D Duvelisib

D.

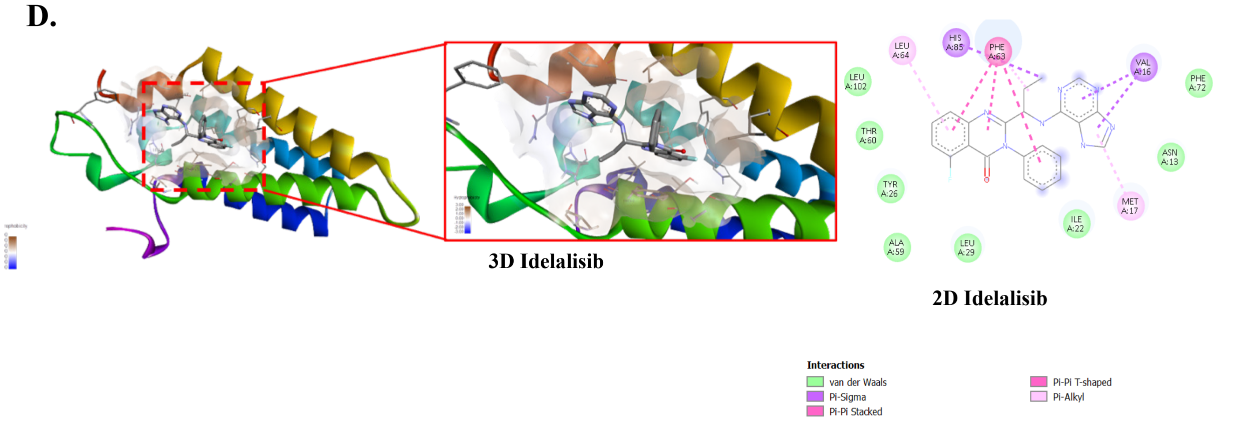

3D Idelalisib

2D Idelalisib

E.

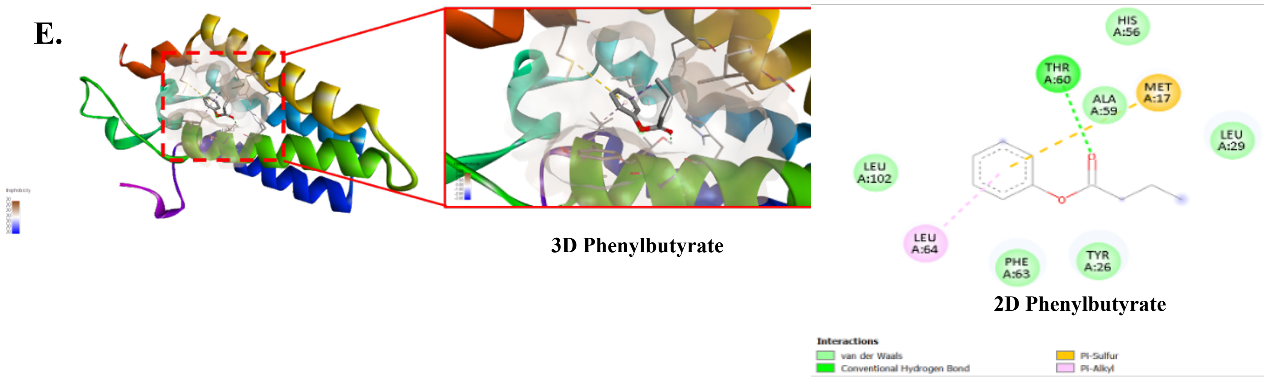

F.

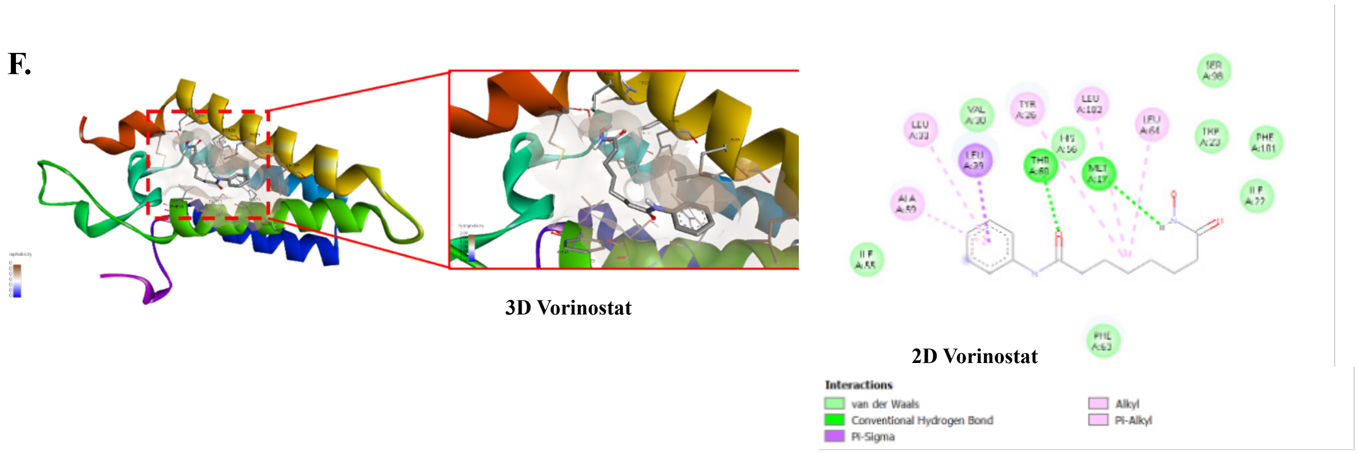

Supplementary Data S4: 3D and 2D molecular binding site of PIK3 inhibitor (https://doi.org/10.1007/s00204-023-03440-4) and HDAC inhibitor (https://doi.org/10.3389/fonc.2020.560487) with ORMDL2. (A) Alpelisib (-8.0 Kcal/Mol). (B) Copanlisib (-6.4 Kcal/Mol). (C) Duvelisib (-7.5 Kcal/Mol). (D) Idelalisib (-7.2 Kcal/Mol). (E) Phenylbutyrate (-5.4 Kcal/Mol). (F). Vorinostat (-7.3 Kcal/Mol).

A.

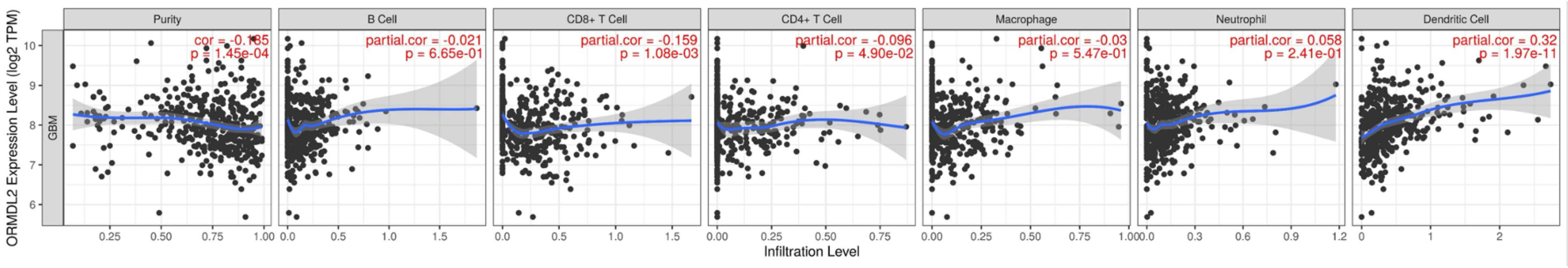

B.

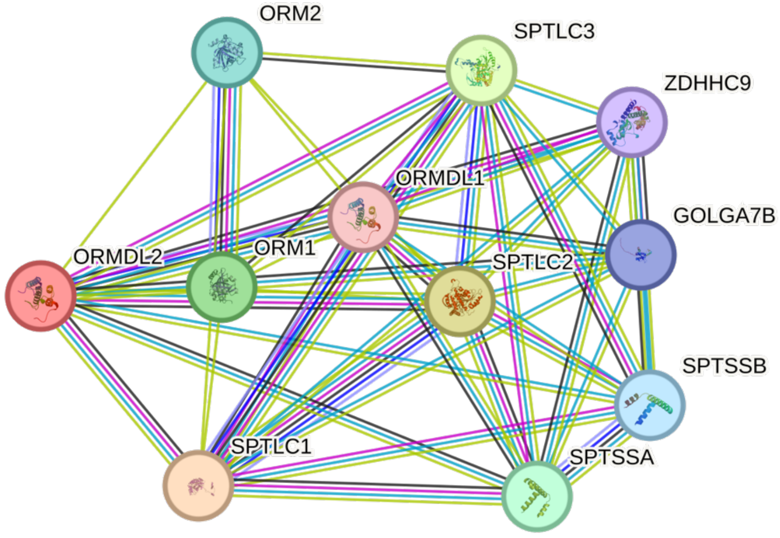

C.

Gene/Protein	Full Name	Function/Role	Cellular Location	Score
ORMDL1	ORMDL Sphingolipid Biosynthesis Regulator 1	Negative regulator of SPT; controls sphingolipid biosynthesis	ER membrane	0.586
SPTLC1	Serine Palmitoyltransferase Long Chain Base Subunit 1	Structural subunit of SPT; stabilizes the complex	ER membrane (cytoplasmic side)	0.969
SPTLC2	Serine Palmitoyltransferase Long Chain Base Subunit 2	Catalytic subunit of SPT; initiates sphingolipid synthesis	ER membrane (cytoplasmic side)	0.946
SPTLC3	Serine Palmitoyltransferase Long Chain Base Subunit 3	Alternative catalytic subunit; alters substrate specificity	ER membrane	0.944
SPTSSA	Serine Palmitoyltransferase Small Subunit A	Enhances SPT activity and substrate binding	ER membrane	0.825
SPTSSB	Serine Palmitoyltransferase Small Subunit B	Regulates SPT activity; may influence substrate specificity	ER membrane	0.735
ORM1	Orosomucoid 1	Acute-phase protein; modulates immune response	Secreted (plasma/serum); Golgi/ER	0.847
ORM2	Orosomucoid 2	Similar to ORM1; involved in inflammation	Secreted; Golgi/ER pathway	0.82
ZDHHC9	Zinc Finger DHHC-Type Palmitoyltransferase 9	Adds palmitate to proteins; regulates membrane association	Golgi and ER membranes	0.61
GOLGA7B	Golgin A7 Family Member B	Golgi-associated protein; involved in trafficking	Golgi apparatus	0.686

Supplementary Data S5: Correlation between ORMDL2 expression and immune cell infiltration in GBM. (A) Scatter plots show the relationship between ORMDL2 expression (log2 TPM) and the infiltration levels of various immune components in glioblastoma multiforme (GBM), including tumor purity, B cells, $CD8^+$ T cells, $CD4^+$ T cells, macrophages, neutrophils, and dendritic cells. Blue lines indicate the trend of correlation (with 95% confidence interval shaded in gray). The Spearman partial correlation coefficient (partial.cor) and p-value are shown in red text in each panel. Notably, ORMDL2 expression shows a significant positive correlation with dendritic cell infiltration (partial.cor = 0.32, p = 1.97e−11), and a significant negative correlation with $CD8^+$ T cells (partial.cor = −0.159, p = 1.08e−03) and tumor purity (cor = −0.185, p = 1.45e−04). These findings suggest that ORMDL2 may be involved in modulating the immune microenvironment in GBM. (B). Protein–protein interaction (PPI) network illustrating the interactome of ORMDL2 generated using STRING analysis. Nodes represent proteins, and edges indicate predicted or known functional associations. ORMDL2 and its interacting partners, including key components of the serine palmitoyltransferase (SPT) complex (SPTLC1/2/3, SPTSSA, SPTSSB), zinc finger palmitoyltransferase (ZDHHC9), and Golgi-associated proteins (GOLGA7B), are shown. (C) Table summarizing gene/protein names, full names, annotated functional roles, subcellular localization, and STRING confidence scores for ORMDL2 and its interactors. ORMDL2, located at the ER membrane, acts as a negative regulator of SPT activity and sphingolipid biosynthesis, which is implicated in GBM pathophysiology.

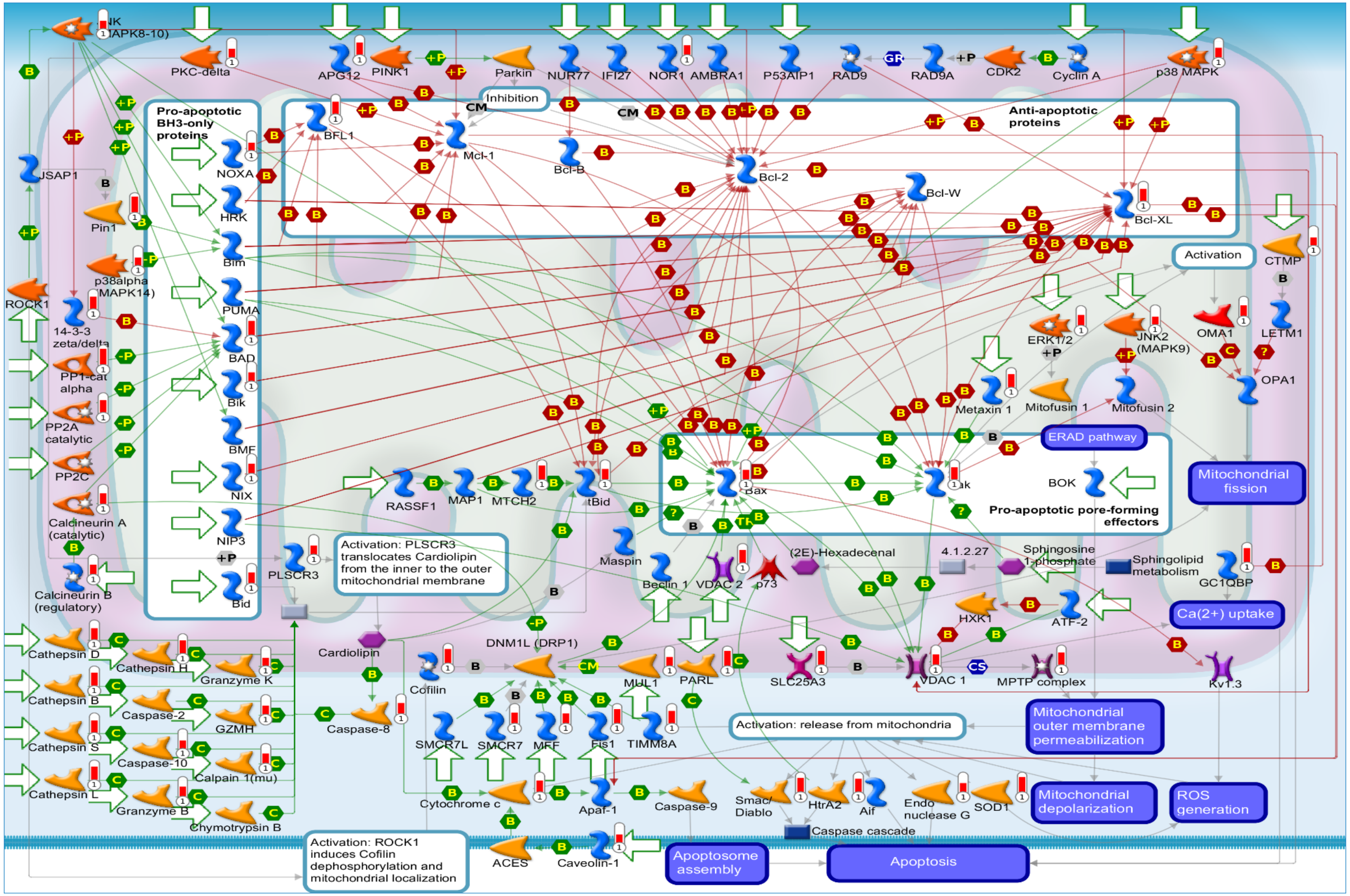

Supplementary Figure S6. MetaCore pathway analysis of ORMDL2 co-expressed genes in GBM patients from TCGA. The " Apoptosis and survival_Regulation of apoptosis by mitochondrial proteins.png" is highlighted, with symbols representing proteins and arrows indicating protein interactions (green for activation and red for inhibition). Thermometer-like histograms visually represent microarray gene expressions, with blue indicating downregulation and red indicating upregulation.

Supplementary Table 1. Top pathways from Gene Set Enrichment Analysis (GSEA)

Name	Size	ES	NES	Nom p-val	FDR q-val	FWER p-val
**GOMF_PRIMARY_ACTIVE_TRANSMEMBRANE_TRANSPORTER_ACTIVITY**	151	0.5830	2.1945	0.0000	0.0040	**0.0040**
**GOMF_ANTIOXIDANT_ACTIVITY**	83	0.6043	2.1503	0.0000	0.0072	**0.0140**
**GOMF_ELECTRON_TRANSFER_ACTIVITY**	104	0.7431	2.1453	0.0000	0.0055	**0.0160**
**GOMF_PROTON_TRANSMEMBRANE_TRANSPORTER_ACTIVITY**	135	0.5682	2.1206	0.0000	0.0063	**0.0240**
**GOCC_FICOLIN_1_RICH_GRANULE_LUMEN**	123	0.6392	2.1576	0.0000	0.0095	**0.0090**
**GOCC_FICOLIN_1_RICH_GRANULE**	184	0.6133	2.1200	0.0000	0.0100	**0.0190**
**GOCC_PEPTIDASE_COMPLEX**	119	0.5680	2.1082	0.0000	0.0085	**0.0210**
**GOCC_PROTON_TRANSPORTING_TWO_SECTOR_ATPASE_COMPLEX_PROTON_ TRANSPORTING_DOMAIN**	24	0.8737	2.0628	0.0000	0.0124	**0.0300**
**GOCC_ORGANELLE_ENVELOPE_LUMEN**	88	0.6181	2.0449	0.0000	0.0129	**0.0390**
**GOCC_OXIDOREDUCTASE_COMPLEX**	116	0.6946	2.0335	0.0000	0.0122	**0.0420**
**GOCC_ENDOPEPTIDASE_COMPLEX**	84	0.6250	2.0326	0.0019	0.0106	**0.0420**
**GOCC_LATE_ENDOSOME_MEMBRANE**	168	0.5061	2.0229	0.0000	0.0114	**0.0490**
**GOBP_ATP_METABOLIC_PROCESS**	126	0.7058	2.1538	0.0000	0.1223	**0.0430**
**HALLMARK_ADIPOGENESIS**	199	0.5290	2.0480	0.0000	0.0200	**0.0120**
**HALLMARK_FATTY_ACID_METABOLISM**	158	0.5380	2.0450	0.0000	0.0100	**0.0130**

**HALLMARK_REACTIVE_OXYGEN_SPECIES_PATHWAY**	49	0.6440	1.9990	0.0000	0.0120	**0.0220**
**HALLMARK_OXIDATIVE_PHOSPHORYLATION**	**200**	**0.7280**	**1.9790**	**0.0040**	**0.0120**	**0.0290**

Supplementary Table 2: Pathway analysis of genes co-expressed with ORMDL2 from the MetaCore database (with p < 0.05 set as the cutoff value).

#	Maps	pValue	Network Objects from Active Data
**1**	Immune response_Antigen presentation by MHC class I: cross-presentation	1.55E-20	Cathepsin L, CLEC12A, Dectin-1, Rab-4A, Rab-27A, FCGRT, VAMP8, LLIR, Cytochrome b-558, Fc epsilon RI gamma, MSR1, Endoplasmin, gp91-phox, NADPH oxidase, HSP90 alpha, FCGR3A, Rac2, Rab-32, p67-phox, Fc gamma RII alpha, Cathepsin S, VAV-1, Cathepsin B, p47-phox, Immunoproteasome (20S core), SEC22B, Syk, Fc gamma RI, IP-30, TIM-3, DAP12, Rab-7, IRAK4, CD74, Rab-11A, C1q, UFO, cPLA2, TLR4, Rab-10, OLR1, Calreticulin, Connexin 43, MHC class I, TLR7, CD40(TNFRSF5), Proteasome (20S core), HSP60, HSP90, CHIP, HSP70, Rab8B, TLR3, SNAP-23, Rab-34, TLR2
**2**	Apoptosis and survival_Regulation of apoptosis by mitochondrial proteins	2.94E-20	Cathepsin L, p38alpha (MAPK14), NIX, PLSCR3, Calcineurin A (catalytic), NOR1, MPTP complex, SMCR7, GC1QBP, PKC-delta, Fis1, Cathepsin H, Granzyme B, 14-3-3 zeta/delta, ERK1/2, MUL1, BFL1, Pin1, PP2A catalytic, PARL, VDAC 2, Bik, Bak, Cytochrome c, Caspase-8, Cathepsin S, MFF, Metaxin 1, Endonuclease G, Cathepsin B, BAD, GZMH, CTMP, Bax, OMA1, Caspase-10, SOD1, SLC25A3, TIMM8A, tBid, PP1-cat alpha, Bcl-XL, MTCH2, NOXA, HtrA2, VDAC 1, JNK(MAPK8-10), JNK2(MAPK9), Caveolin-1, Calpain 1(mu), Calcineurin B (regulatory), Smac/Diablo, p38 MAPK, APG12, Cathepsin D, Granzyme K, Cofilin, Bid
**3**	Dengue virus infection mechanism	1.37E-13	IRp60, GRP78, Calcineurin A (catalytic), REA, TRAF3, CD44, STAM1, IL-10, Fc epsilon RI gamma, ATP6V0D1, ERK1/2, TBK1, Prohibitin, RP40, TIM-4, HSP90 alpha, FCGR3A, TANK, Fc gamma RII alpha, IRF1, Rnasek, Rac1, RhoGDI alpha, Syk, IL-1 beta, Fc gamma RI, TSA-1, ILT2, DAP12, Rab-7, CD14, UFO, APG5, CARD5, SOCS3, MDL-1, VAS1, CDC42, SHP-1, TLR7, HSP70, Caspase-1, TLR3, Protein S, Calcineurin B (regulatory), APG12, LAMR1
**4**	COVID-19: immune dysregulation	2.2E-13	GRO-2, TGF-beta 1, HLA-DRB1, MHC class II, UNC93B, IL-1 alpha, IP10, IL-18, CCL2, IL-10, IL-8, Granzyme B, CCR2, HLA-DMA, HLA-DPA1, MIP-1-beta, HLA-DRA1, CXCL16, IFN-gamma receptor, IL1RN, IRF1, Leptin receptor, HLA-DMB, Granzyme A, Btk, Perforin, CCL7, CCL5, IL-1 beta, TIM-3, HLA-E, IRAK4, CCL8, FasR(CD95), CD4, MHC class I, IL-6, IL-10 receptor, CCR5, TLR7, TLR8, LDHA, MIP-1-alpha, Caspase-1, TLR3, HLA-DPB1, PKM2
**5**	Immune response_Antigen presentation by MHC class II	2.93E-13	MHC class II alpha chain, Cathepsin L, RhoA, Dectin-1, MHC class II, ARL14EP, FCGRT, HSC70, MAP1LC3B, LLIR, AP complex 2 medium (mu) chain, PKC-delta, Fc epsilon RI gamma, HCLS1, HLA-DM, Endoplasmin, ERK1/2, Fc alpha receptor, NADPH oxidase, HSP90 alpha, FCGR3A, Cathepsin S, Cathepsin V, Rac1, MAP1LC3A, Syk, 14-3-3

			beta/alpha, Cathepsin F, IP-30, Rab-7, CD74, PKC, Alpha-centractin, SPPL2a, RILP (Rab interacting lysosomal protein), Gie1, TLR4, OLR1, LAMP2, CD4, Fc gamma RII beta, CDC42, JNK(MAPK8-10), Legumain, Proteasome (20S core), HSP90, MHC class II beta chain, R-Ras, Dynein 1, cytoplasmic, intermediate chains, p38 MAPK, TLR2, Tubulin (in microtubules)
**6**	Chemokines in inflammation in adipose tissue and liver in obesity, type 2 diabetes and metabolic syndrome	3.23E-13	MHC class II, CD68, CD45, LYVE-1, CD44, CCL2, IL-8, CCR2, FCGR3A, PECAM1, ITGAM, CCL5, IL-1 beta, Fc gamma RI, L-selectin, CD14, CMKLR1, PSGL-1, TLR4, IL-6, MIF, PLAUR (uPAR), CCR5, EMR1, MIP-1-alpha, CD86, CXCR4, CD163, TLR2, CD1c
**7**	Role of microglia in Alzheimer disease	1.09E-12	C5aR, VPS29, FPRL1, Vps26A, alpha-M/beta-2 integrin, IL10RA, CCL2, IL-10, MSR1, Lyn, DAP10, Beta-catenin, MIP-1-beta, APOE, GRO-3, NFKBIA, VAV-1, FPR, Cathepsin B, Rac1, TREM2, Syk, IL-1 beta, DAP12, CD14, C1q, CD33, CARD5, TLR4, IL-6, C1qa, CD40(TNFRSF5), iC3b, MIP-1-alpha, Caspase-1, PLC-gamma 2, p38 MAPK, CD59, CD47, TLR2
**8**	Immune response_HSP60 and HSP70/ TLR signaling	3.3E-12	MHC class II, IL-10, IL-8, ERK1/2, MD-2, E2N(UBC13), CD69, Ubiquitin, IL-1 beta, I-kB, AP-1, IRAK4, CD14, IKK-alpha, TLR4, MEK6(MAP2K6), IKK-gamma, MHC class I, IL-6, MEK1/2, TPL2(MAP3K8), IRAK1/2, CD83, JNK(MAPK8-10), CD40(TNFRSF5), HSP60, UEV1A, HSP70, p38 MAPK, CD86, TLR2
**9**	Immune response_IL-5 signaling via PI3K, MAPK and NF-kB	1.06E-11	RhoA, Syntenin 1, GRB2, alpha-M/beta-2 integrin, ITGB2, CCL2, PKC-delta, IL-8, CSF2RB, Lyn, H-Ras, F-Actin cytoskeleton, ERK1/2, Fc alpha receptor, NADPH oxidase, Beta-catenin, Pin1, Fc gamma RII alpha, PP2A cat (alpha), ITGAM, FADD, LIMK, Btk, NFKBIA, p47-phox, Syk, IL-1 beta, Bax, Unc-119, AP-1, Calpastatin, cPLA2, ICAM3, Bcl-XL, Fc gamma RII beta, MEK1/2, JNK(MAPK8-10), Calpain 1(mu), p38 MAPK, Cofilin, LPCAT2
**10**	Immune response_Induction of the antigen presentation machinery by IFN-gamma	1.21E-11	IFNGR1, HLA-DRB1, MHC class II, PSMB8(LMP7), ASH2L, HLA-DMA, PSMB9, HLA-DPA1, HLA-DOA, HLA-F, IFNGR2, HLA-DRA1, IRF1, USF1, HLA-DQA1, HLA-DMB, Cathepsin S, PSME1, Immunoproteasome (20S core), Beta-2-microglobulin, HLA-E, CD74, PSME2, Nucleophosmin, PSMB10, HLAB, MHC class I, RFXANK, HLAC, HLA-DPB1
**11**	Neutrophil resistance to apoptosis in COPD and proresolving impact of lipid mediators	2.17E-11	FPRL1, alpha-M/beta-2 integrin, TNF-R2 soluble, TNF-R1 soluble, Cytochrome b-558, p22-phox, FasR(CD95) soluble, gp91-phox, ERK1/2, 14-3-3, TRADD, p90RSK1, p67-phox, Bak, Cytochrome c, FADD, Caspase-8, p47-phox, BAD, Bax, Calpastatin, c-IAP1, tBid, MEK6(MAP2K6), FasR(CD95), Bcl-XL, p40-phox, Calpain 1(mu), TNF-R1, Smac/Diablo, p38 MAPK, Bid
**12**	ATP/ITP metabolism	3.21E-11	ITPA, RRP41, NDPK C, POLR3F, NDPK 7, NDPK complex, RPB8, ADA, RPB10, RPB6, RRP40, POLR2G, AMD3, RPA16, POLR2D, RRP42, RRP43, RPB7.0, NDPK 6, ADSL, AMP deaminase, 5'-NT1B, AK3, POLR2C, POLR1B, AK2, CSL4, KAD7, RRP4, PPAP, RRM2B, NDPK A, PPA5, RRP46, ADSS, ACYP2, PNPH, PPAL, NDPK D (mitochondrial), POLR2I, RPA39, POLR2J, Adenosine kinase, ENP1, HPRT, POLR3K, APRT, Small RR subunit, NDPK B, AK1
**13**	Ubiquinone metabolism	4.06E-11	NDUFB9, NDUFB7, COQ6, NDUFA11, NDUFS1, NDUFS3, NDUFAB1, NDUFA6, NDUFC1, NDUFS4, NDUFA2, NDUFB2, NDUFA3, NDUFV2, NDUFB4, NDUFB6, NDUFS6, NDUFB10, NDUFA8, NDUFB3, NDUFS8, DAP13,

			NDUFS5, NDUFB1, NDUFB5, NDUFC2, NDUFV3, NDUFA7, NDUFA9, COQ3, NDUFA13, coenzyme Q2 homolog, prenyltransferase (yeast), NDUFA5, NDUFA1, NDUFA4, NDUFB8
**14**	Immune response_TLR2 and TLR4 signaling	9.03E-11	TLR1, Pellino 3, TLR10, IL-10, IL-8, Lyn, H-Ras, ERK1/2, MD-2, MSK1/2 (RPS6KA5/4), E2N(UBC13), p90Rsk, VAV-1, Rac1, Ubiquitin, IL-1 beta, I-kB, AP-1, Hck, IRAK4, CD14, IKK-alpha, TLR4, IRAK1, MEK6(MAP2K6), IRF5, IKK-gamma, IL-6, MEK1/2, TPL2(MAP3K8), JNK(MAPK8-10), UEV1A, p38 MAPK, TLR2
**15**	Immune response_TREM1 signaling	7.32E-10	MEK1(MAP2K1), Calcineurin A (catalytic), GRB2, CCL2, IL-8, H-Ras, ERK1/2, E2N(UBC13), p90RSK1, NFKBIA, BAD, Syk, Ubiquitin, IL-1 beta, I-kB, Bax, DAP12, IKK-alpha, MALT1, TLR4, PLC-gamma, IKK-gamma, IL-6, TREM1, Bcl-10, MIP-1-alpha, Calcineurin B (regulatory), Elk-1, TLR2, WBSCR5(NTAL)
**16**	Apoptosis and survival_Caspase cascade	2.7E-09	Caspase-5, GSDMDC1, c-IAP2, Caspase-6, Granzyme B, Caspase-4, TRADD, Apo-2L(TNFSF10), Bak, Cytochrome c, FADD, Caspase-8, Gelsolin, Bax, Caspase-10, Caspase-7, c-IAP1, tBid, RAIDD, FasR(CD95), Bcl-XL, HtrA2, VDAC 1, TNF-R1, Caspase-1, TWEAK(TNFSF12), Smac/Diablo, Bid
**17**	Neutrophil chemotaxis in asthma	3.81E-09	C5aR, GRO-2, FPRL1, CCL2, PLGF, IL-8, G-protein alpha-i family, Substance P receptor, CCR2, Tissue kallikreins, ERK1/2, CCR1, GRO-3, CCL7, CCL5, MIF, G-protein beta/gamma, MIP-1-alpha, HSP70, PI3K reg class IB (p101), TLR2, CCL15
**18**	Transport_Clathrin-coated vesicle cycle	5.46E-09	Rab-4, VTI1A, VAMP8, VAMP7, Myosin I, PIP5KIII, Eps15, SAR1A, VTI1B, VAMP4, ARF1, DAB2, Actin, Syntaxin 8, Syntaxin 7, GS15, RAB9P40, RABGEF1, Rab-7, SAR1, Rab-11A, Endophilin B1, RILP (Rab interacting lysosomal protein), Syntaxin 5, Rab-9, Syntaxin 12, PREB, BIN1 (Amphiphysin II), TIP47, GDI2, Actin cytoskeletal, Optineurin
**19**	Protein folding and maturation_POMC processing	5.48E-09	proACTH, POMC, N-POMC, gamma-LPH, Joining peptide (JP), gamma-MSH, beta-MSH, gamma3-MSH, ACTH, PAM, NAT-1, beta-Endorphin, ACTH 1-17, DA-alphaMSH, beta-LPH, gamma2-MSH, N-POC, alpha-MSH, CLIP
**20**	Signal transduction_MIF signaling	6.24E-09	PU.1, Heme oxygenase 1, CD44, IL-8, Lyn, GCL reg, G-protein alpha-i family, ERK1/2, PRDX1, CD74-ICD, BAD, Syk, AP-1, CD74, SPPL2a, cPLA2, TLR4, ACKR3, Bcl-XL, MIF, MEK1/2, G-protein beta/gamma, JNK(MAPK8-10), Beta-arrestin1, NRF2, SFK, PI3K reg class IB (p101), NQO1, CXCR4
**21**	HSP70 and HSP40-dependent folding in Huntington's disease	7.16E-09	HSC70, HSP90 alpha, Hdj-2, Sti1, Ubiquitin, PSMD1, DNAJB6 (Hdj-1), ST13 (Hip), SGTB, HSP27, HSP40, Proteasome (20S core), HSP90, BAG-1, CHIP, HSP70, Cathepsin D
**22**	Apoptosis and survival_Apoptotic TNF-family pathways	7.36E-09	TNF-R2, LTBR(TNFRSF3), TRAF3, CD70(TNFSF7), TRADD, Apo-2L(TNFSF10), FADD, Caspase-8, Bax, Caspase-10, Caspase-7, c-IAP1, tBid, RAIDD, FasR(CD95), NADE(NGFRAP1), SIVA1, CD27(TNFRSF7), Bcl-XL, TNF-R1, TWEAK(TNFSF12), Smac/Diablo, Bid
**23**	Regulation of degradation of CFTR in cystic fibrosis	7.43E-09	RNF5, UFD1, HSC70, Hdj-2, Sti1, E2I, DNAJB12, SUMO-3, Ubiquitin, RNF4, Aha1, SUMO-2, HSP27, UCHL1, BAG-2, UBE2D1, Proteasome (20S core), HSP90, CHIP, HSP70, Dynein 1, cytoplasmic, intermediate chains, Derlin1

**24**	GTP-XTP metabolism	7.98E-09	ITPA, RRP41, NDPK C, POLR3F, NDPK 7, NDPK complex, RPB8, KGUA, RPB10, RPB6, RRP40, POLR2G, RPA16, POLR2D, RRP42, RRP43, RPB7.0, NDPK 6, 5'-NT1B, POLR2C, POLR1B, CSL4, RRP4, NDPK A, PPA5, RRP46, NDPK 8, PNPH, PPAL, NDPK D (mitochondrial), POLR2I, RPA39, POLR2J, GMP2, HPRT, POLR3K, NDPK B
**25**	Immune response_Fc epsilon RI pathway: Lyn-mediated cytokine production	1.03E-08	Calcineurin A (catalytic), Slp76, GRB2, CCL2, PKC-delta, Fc epsilon RI gamma, Lyn, H-Ras, ERK1/2, MEF2C, BFL1, TSLP, GRAP2, SKAP55, Btk, NFKBIA, VAV-1, Rac1, Fc epsilon RI alpha, Syk, IL-1 beta, AP-1, PKC, MALT1, PLC-gamma, MEK6(MAP2K6), MAP2K5 (MEK5), IKK-gamma, IL-6, MEK1/2, CDC42, JNK(MAPK8-10), Bcl-10, FYB1, p38 MAPK, Elk-1
**26**	NETosis in SLE	1.2E-08	Histone H3, IL-18, ERK1/2, NADPH oxidase, Pin1, p67-phox, Fc gamma RII alpha, Histone H2, p47-phox, IL-1 beta, PKC, C1q, TLR7, Alpha-defensin, Histone H2A, p38 MAPK, Histone H1.2, Histone H4, Histone H1
**27**	Apoptosis and survival_TNFR1 signaling	1.34E-08	c-IAP2, Caspase-6, jBid, TRADD, Cytochrome c, FADD, Caspase-8, GCK(MAP4K2), I-kB, Bax, Caspase-10, Caspase-7, BRE, c-IAP1, tBid, RAIDD, IKK-gamma, HtrA2, c-FLIP(Short), JNK(MAPK8-10), TNF-R1, Smac/Diablo, Bid
**28**	Role of Apo-2L(TNFSF10) in Prostate Cancer cell apoptosis	1.35E-08	c-IAP2, c-FLIP(Long), TRADD, Apo-2L(TNFSF10), Bak, Cytochrome c, FADD, Caspase-8, I-kB, Bax, Caspase-10, Caspase-7, c-IAP1, tBid, IKK-gamma, Bcl-XL, Osteoprotegerin, Smac/Diablo, TRID(TNFRSF10C), Bid
**29**	CTP/UTP metabolism	1.47E-08	ITPA, RRP41, NDPK C, POLR3F, PCY1A, NDPK 7, UDP, NDPK complex, KCY, RPB8, RPB10, RPB6, RRP40, POLR2G, RPA16, POLR2D, NT5M, RRP42, RRP43, UCK1, RPB7.0, NDPK 6, AK3, POLR2C, POLR1B, AK2, CSL4, RRP4, DPYD, NDPK A, RRP46, NDPK D (mitochondrial), PD-ECGF (TdRPase), POLR2I, CDD, RPA39, POLR2J, ENP1, POLR3K, NDPK B, AK1
**30**	Apoptosis and survival_Role of PKR in stress-induced apoptosis	1.66E-08	TRAM, C/EBP zeta, TRAF3, ATF-3, eIF4E, ERK1/2, ATF-4, PP2A regulatory, IFN-gamma receptor, PP2A catalytic, p21, IRF1, Caspase-8, NFKBIA, PACT, 4E-BP1, I-kB, Caspase-7, IKK-alpha, TARBP2, TLR4, FasR(CD95), eIF2S1, MSK2, TNF-R1, TLR3
**31**	Maturation and migration of dendritic cells in skin sensitization	2.58E-08	MHC class II alpha chain, TNF-R2, HLA-DRB1, MHC class II, IL-8, ERK1/2, HLA-DRB3, HLA-DRA1, TRADD, GCK(MAP4K2), IL-1 beta, I-kB, HLA-DRB, MEK6(MAP2K6), IL-6, CD83, JNK(MAPK8-10), CD40(TNFRSF5), MHC class II beta chain, TNF-R1, p38 MAPK, CD86
**32**	Macrophage and dendritic cell phenotype shift in cancer	8.65E-08	IFNGR1, TGF-beta 1, MHC class II, IP10, alpha-M/beta-2 integrin, IL-4R type I, PLGF, IL-10, MSR1, XBP1, M-CSF receptor, IL-4R type II, Apo-2L(TNFSF10), ITGAM, ILT4, IL-1 beta, I-kB, GM-CSF receptor, TLR4, SOCS3, IRF5, IL-1RI, IKK-gamma, MHC class I, IL-6, IL-10 receptor, CD137 ligand(TNFSF9), SHP-1, TLR7, CD40(TNFRSF5), STAT6, TNF-R1, Calcineurin B (regulatory), p38 MAPK, CD86, ILT3, TLR2, TGF-beta receptor type I
**33**	Immune response_Sublytic effects of membrane attack complex	1.26E-07	RhoA, TGF-beta 1, GRP78, GRB2, C/EBPbeta, Endoplasmin, G-protein alpha-i family, H-Ras, ERK1/2, c-FLIP(Long), MNK1, IRF1, Caspase-8, BAD, MAPKAPK2, PKC, cPLA2, HSP27, MEK6(MAP2K6), IL-6, Bcl-XL, MEK1/2, G-protein beta/gamma, Actin cytoskeletal, eIF2S1, Pnpla8, PI3K reg class IB (p101), RGC32, p38 MAPK

**34**	Immune response_Antigen presentation by MHC class I, classical pathway	1.33E-07	PDIA3, MIC2, PSMB8(LMP7), Endoplasmin, PSMB1, PSMB5, PSMB9, HSP90 alpha, PA28 (11S regulator), SPC, Impas 1, PSME1, Immunoproteasome (20S core), MHC Class I alpha chain, Beta-2-microglobulin, PSME2, PSMB10, Calreticulin, PSMB2, MHC class I, TAPBPL, Proteasome (20S core), CHIP, HSP70, BCAP31
**35**	Role of TLR signaling in skin sensitization	1.36E-07	TRAM, TRAF3, TBK1, IKK-epsilon, IL-1 beta, I-kB, AP-1, IRAK4, CD14, IKK-alpha, HSP27, TLR4, MEK6(MAP2K6), IKK-gamma, IL-6, IRAK1/2, JNK(MAPK8-10), CD40(TNFRSF5), HSP70, p38 MAPK, CD86, TLR2
**36**	Role of integrins in eosinophil degranulation in asthma	1.68E-07	Galectin-3, alpha-M/beta-2 integrin, CSF2RB, C3b, G-protein alpha-i family, Substance P receptor, ERK1/2, FGR, Fc alpha receptor, Eotaxin-3, RNS2, FCGR3A, CD9, Fc gamma RII alpha, ITGAM, Plastin, CCL7, CCL5, Hck, GM-CSF receptor, PKC, CCL13, MEK1/2, CysLT1 receptor, ECP (RNase 3), p38 MAPK
**37**	Impaired macrophage phagocytic function in asthma	1.71E-07	RhoA, TNF-R2, SP-A, GSHB, alpha-M/beta-2 integrin, GCL reg, FCGR3A, ITGAM, Rac1, GSTP1, Fc gamma RI, TIM-3, CD14, SOD1, Calreticulin, ICAM3, PA24A, iC3b, TNF-R1, NRF2
**38**	Immune response _CCR3 signaling in eosinophils	2.26E-07	MLCP (cat), Profilin I, MEK1(MAP2K1), RhoA, Cytochrome b-558, p22-phox, G-protein alpha-i family, H-Ras, gp91-phox, FGR, Eotaxin-3, Rac2, p67-phox, p47-phox, Rac1, CCL7, CCL5, Hck, Profilin, CCL13, CCL8, cPLA2, MRLC, G-protein beta/gamma, WASP, Actin cytoskeletal, LIMK2, MELC, PI3K reg class IB (p101), p38 MAPK, Cofilin
**39**	Immune response_CD40 signaling in B cells	2.57E-07	c-IAP2, TRAF3, H-Ras, ALOX5, ERK1/2, BLNK, ALOX5AP, NADPH oxidase, BFL1, IRF1, Btk, NFKBIA, Rac1, IKK-epsilon, Syk, Cyclin D2, I-kB, AP-1, MAPKAPK2, IKK-alpha, c-IAP1, FasR(CD95), IL-6, Bcl-XL, MEK1/2, TPL2(MAP3K8), JNK(MAPK8-10), CD40(TNFRSF5), p40-phox, Dynein 1, cytoplasmic, intermediate chains, PLC-gamma 2, p38 MAPK, CXCR4
**40**	Tau dysregulation in Alzheimer disease	2.57E-07	G3P2, Calcineurin B1, CDK5, Calcineurin A (catalytic), HSC70, Caspase-6, SUMO-1, p38delta (MAPK13), C/EBPbeta, RPS6, PP1-cat, ERK1/2, PP2A regulatory, PP2A catalytic, Calcipressin 1, Alpha-2A adrenergic receptor, PP2A cat (alpha), Caspase-8, PPP2R2A, p70 S6 kinases, RBBP7 (RbAp46), JKAP, MEK6(MAP2K6), BIN1 (Amphiphysin II), LCMT1, JNK(MAPK8-10), Beta-2 adrenergic receptor, Legumain, PRNP, HSP90, Calpain 1(mu), CHIP, Calcineurin B (regulatory)
**41**	Immune response_CD40 signaling in dendritic cells, monocytes, and macrophages	3.19E-07	Sequestosome 1(p62), c-IAP2, MHC class II, IL-1 alpha, TRAF3, IL-10, PKC-delta, IL-8, Lyn, H-Ras, ERK1/2, NFKBIA, VAV-1, Syk, IL-1 beta, p70 S6 kinases, IKK-alpha, N-Ras, MEK6(MAP2K6), IL-1RI, MHC class I, IL-6, Bcl-XL, MEK1/2, TPL2(MAP3K8), CD83, JNK(MAPK8-10), JNK2(MAPK9), CD40(TNFRSF5), p38 MAPK, CD86
**42**	Oxidative stress_Role of Sirtuin1 and PGC1-alpha in activation of antioxidant defense system	3.82E-07	SCPX, Sequestosome 1(p62), PRDX3, Thioredoxin, Heme oxygenase 1, GSHB, GCL reg, MT-TRX, AMPK beta subunit, MSRA, Esrra, AMPK gamma subunit, UCP2, PRDX1, GSHR, GSTP1, PRDX5, GPX1, SOD1, Sestrin 1, PBEF, Catalase, TXNRD1, NRF2, NQO1, PPAR-gamma
**43**	Cell adhesion_Integrin inside-out signaling in neutrophils	4.46E-07	MEK1(MAP2K1), Slp76, alpha-M/beta-2 integrin, PREL1, ITGB2, CD44, URP2, PKC-delta, IL-8, Lyn, G-protein alpha-i family, FGR, Btk, G-protein alpha-i2, FPR, Rac1, Syk, RAP-1A, Fc gamma RI, L-selectin, DAP12, Hck, VASP, PSGL-1, MEK1/2, G-protein beta/gamma, FYB1, Cytohesin1, PI3K reg class IB (p101), PLC-gamma 2, p38 MAPK

**44**	The complement system and macrophages in neuropathic pain	4.46E-07	C5aR, p38alpha (MAPK14), TNF-R2, TGF-beta 1, Factor B, GC1QBP, CCL2, PKC-delta, C3, C3b, G-protein alpha-i family, Substance P receptor, CCR2, ERK1/2, CCR1, G-protein alpha-q/11, Angiotensin II, C1qb, C3 convertase (C3bBb), Factor D, C1qc, IL-1 beta, C1, C1q, TLR4, Factor Bb, IL-1RI, C2, DAF, IL-6, G-protein beta/gamma, CCR5, MIP-1-alpha, TNF-R1, C3a, C5 convertase (C3b2Bb), p38 MAPK, ERK1 (MAPK3), C3aR, TLR2, C2a
**45**	Immune response_Role of PKR in stress-induced antiviral cell response	4.83E-07	p38alpha (MAPK14), TRAF3, IL-10, IL-8, ERK1/2, IFN-gamma receptor, IRF1, Caspase-8, NFKBIA, PACT, IL-1 beta, I-kB, Caspase-7, IKK-alpha, TARBP2, TLR4, MEK6(MAP2K6), IL-1RI, IL-6, JNK(MAPK8-10), MSK2, TNF-R1, TLR3, TLR2, BAFF(TNFSF13B)
**46**	Immune response_IFN-gamma in macrophages activation	4.87E-07	PU.1, IRF8, IP10, IL-18, CCL2, IL-10, HAF1, C/EBPbeta, gp91-phox, IFN-gamma receptor, p67-phox, IRF1, C1qb, C1qc, CCL5, Fc gamma RI, DEPTOR, TLR4, Selenoprotein P, IL-6, CD40(TNFRSF5), Caspase-1, PPAR-gamma
**47**	IFN-gamma and Th2 cytokines-induced inflammatory signaling in normal and asthmatic airway epithelium	5.07E-07	IP10, CCL2, IL-4R type I, IL-8, CXCR6, IL-4R type II, ERK1/2, Eotaxin-3, CXCL16, IFN-gamma receptor, IL4RA, IRF1, TSLP, CCL5, SOCS3, IL-2R gamma chain, IL-6, JNK(MAPK8-10), STAT6, p38 MAPK
**48**	Immune response_Down-regulation of mast cell functions through ITIM-containing inhibitory receptors	5.75E-07	IRp60, CCL2, Fc epsilon RI gamma, Lyn, ERK1/2, KLRG1, PECAM1, Csk, PIRB, c-Kit, DOK2, Fc epsilon RI alpha, Syk, CD72, LAIR1, MHC class I, DOK1, SHP-1, CD47
**49**	Apoptosis and survival_p53 and p73-dependent apoptosis	6.42E-07	Cathepsin L, NIX, NOR1, Caspase-6, PLEKHF1, Glyoxalase II, PKC-delta, Prohibitin, Pin1, Apo-2L(TNFSF10), IL4RA, Bik, Bak, BAD, Cystatin C, MM-1, ZNHIT1, Bax, Caspase-10, Caspase-7, GPX1, FasR(CD95), MDM2, SIVA1, Bcl-XL, GADD45 alpha, NOXA, HtrA2, Caspase-1, p38 MAPK, Cathepsin D, Bid
**50**	Immune response_C3a signaling	9.14E-07	RhoA, TGF-beta 1, MHC class II, CCL2, KLF5, IL-8, G-protein alpha-i family, ERK1/2, MIP-1-beta, NFKBIA, CCL5, IL-1 beta, AP-1, IKK-alpha, IL-6, MEK1/2, G-protein beta/gamma, CD40(TNFRSF5), C3a, PI3K reg class IB (p101), p38 MAPK, Elk-1, CD86, C3aR

Supplementary Table 3: Top ranks drug docking based on binding affinity

No.	Drug target	Binding affinity (Kcal/Mol)
1.	Umbralisib	-8.6*
2.	Lapatinib	-8
3.	Alpelisib	-8
4.	Afatinib	-7.6
5.	Duvelisib	-7.5
6.	Vorinostat	-7.3
7.	Idelalisib	-7.2
8.	Copanlisib	-6.4
9.	Temozolomide	-5.5
10.	Phenlybyutarate	-5.4
